# Proper irrigation amount for eggplant cultivation in a solar greenhouse improved plant growth, fruit quality and yield by influencing the soil microbial community and rhizosphere environment

**DOI:** 10.3389/fmicb.2022.981288

**Published:** 2022-09-23

**Authors:** Tuo Ji, Xinyong Guo, Fengling Wu, Min Wei, Jing Li, Ping Ji, Ningxin Wang, Fengjuan Yang

**Affiliations:** ^1^State Key Laboratory of Crop Biology, College of Horticulture Science and Engineering, Shandong Agricultural University, Tai’an, Shandong, China; ^2^Scientific Observing and Experimental Station of Facility Agricultural Engineering (Huang-Huai-Hai Region), Ministry of Agriculture and Rural Affairs, Tai’an, Shandong, China; ^3^Shandong Collaborative Innovation Center for Fruit and Vegetable Production with High Quality and Efficiency, Tai’an, Shandong, China; ^4^Key Laboratory of Biology and Genetic Improvement of Horticultural Crop (Huang-Huai Region), Ministry of Agriculture and Rural Affairs, Tai’an, Shandong, China; ^5^School of Economics, Qingdao University, Qingdao, Shandong, China; ^6^College of Plant Protection, Shandong Agricultural University, Tai’an, Shandong, China

**Keywords:** irrigation amount, microbial community, soil physicochemical factors, yield, eggplant

## Abstract

Water scarcity is a worldwide problem, and in order to obtain plenty of production, agricultural irrigation water accounts for a large portion. Many studies have shown that the interaction of root microorganisms and soil can promote crop growth. Developing ways to reduce irrigation to maintain soil fertility and ensure crop yield by regulating the root microenvironment is an important research goal. Here, we developed a reasonable irrigation plan for eggplant cultivation in a solar greenhouse. The maximum theoretical amount of water demand during eggplant planting obtained from a previous study was used as the control (CK), and the irrigation in the treatments was reduced by 10, 20 and 30% relative to this amount. The 10% irrigation reduction treatment (T1) significantly improved soil nutrients and increased soil catalase, urease and alkaline phosphatase activities (*p* < 0.05). Further analysis of rhizosphere microorganisms revealed the highest richness and diversity of the microbial community under the T1 treatment, with Bacilli as the most abundant bacteria and Aspergillaceae as the most abundant fungi and lower relative abundances of Chloroflexi and Acidobacteria (*p* < 0.05). Changes in microbial community structure under the influence of different irrigation treatments resulted in improvements in rhizosphere N cycling and nutrient catabolism. The plant–microbe interactions led to significant increases in eggplant plant height, root vigour, root surface area, leaf chlorophyll a, leaf net photosynthetic rate, water use efficiency, transpiration rate, and stomatal conductance under the T1 treatment compared to the CK treatment; soluble sugar, soluble protein and free amino acid contents in eggplant fruit increased by 10.8, 12.3 and 6.7%, respectively; and yield increased by 3.9%. Our research proved that the 10% irrigation reduction treatment (T1) could improve microbial community richness and fruit yield, which would improve irrigation efficiency and cost reduction in agriculture.

## Introduction

Water scarcity is a major challenge in agriculture. As the population grows and agricultural production expands, the demand for agricultural irrigation water is constantly increasing. However, the anticipation of a climate change and drought has made researchers less optimistic in their predictions for future agricultural water supplies (Ault Toby, 2020; Berdugo et al., 2020). To face the challenge of producing more food in the coming decades, it is necessary to improve the water use efficiency (WUE) in agricultural irrigation (Du et al., 2015).

For crops, especially vegetables, an adequate water supply is essential, but this does not mean that more water is better. Excessive water can cause stress similar to that caused by drought and even plant death (Hirabayashi et al., 2013). Flooding causes hypoxia in plant roots, which can cause irreversible damage (Voesenek and Bailey-Serres, 2015). The amount of irrigation determines the maintenance of nutrients in the soil, which affects the growth and development of plants. Agricultural producers need more water-efficient cropping systems to cope with high water use in irrigated agriculture and high unproductive losses due to runoff and evaporation (Bodner et al., 2015). Among them, water-saving irrigation measures are crucial. Studies have found that a proper reduction in agricultural irrigation is not inevitably detrimental to crops but instead benefits crops in terms of photosynthetic metabolism (Morales et al., 2020). Alternate drip irrigation of 60% field capacity showed no significant difference of tomato yields compared to conventional surface drip irrigation (Wang et al., 2020). The alternate wetting and drying irrigation method, which reduced the total irrigation amount, improved rice grain yield compared with continuous flooding without reducing the quality of grains (Song et al., 2021).

Different irrigation regulation strategies have also been applied in agricultural production, such as regulated deficit irrigation (RDI; Pérez-Álvarez et al., 2021), partial root-zone drying (PRD; Santos et al., 2021) and alternate partial root-zone irrigation (APRI; Kang and Zhang, 2004). Studies on eggplants in greenhouse found that APRI increased the fruit yield and WUE (Du et al., 2005). The same results were found in cucumber research (Fang et al., 2015). However, most studies have focused on the relationship involving yield, quality of the eggplant and irrigation, but few studies have focused on the changes in the soil’s physical and chemical properties caused by different irrigation amounts and their effect on root microorganisms.

The interaction between plant roots and soil microorganisms affects plant growth, including root morphology, the root-to-shoot weight ratio, mineral content and uptake, and the rate of development, thus influencing crop yield and physiological processes (Rovira, 1965). Soil water content has a great impact on microbial communities and affects soil nutrients and pH, which in turn affects the growth and development of plants (Girvan Martina et al., 2003; Frey et al., 2004; Faoro et al., 2010; Rousk et al., 2010). Drought will promote an increase in specific root exudation, which affects soil microorganisms (Naylor and Coleman-Derr, 2018; Williams and de Vries, 2020). Water deficit increased the ratio of Gram-positive to Gram-negative bacteria (Fuchslueger et al., 2014, 2016; Chodak et al., 2015) and increased the proportion of actinobacteria (Hartmann et al., 2017). The reasons for these changes include differences in the substrate preference and metabolic capacity among different microorganisms, the tolerance of microorganisms to water deficit, and the activity level of specific bacteria (Naylor and Coleman-Derr, 2018). Wheat inoculated with inactivation of non-ribosomal peptide and polyketide derived metabolites *P. polymyxa* bacteria resulted in two times higher plant survival and three times increased biomass under severe drought stress, compared to wild type (Timmusk et al., 2015). Inoculation of *Pseudomonas* sp. strain GAP-P45 increased the survival, plant biomass, and root adhering soil/root tissue ratio of sunflower seedlings under drought stress (Sandhya et al., 2009). There are also studies suggesting that plant growth-promoting rhizosphere bacteria can produce plant hormones, affect cell division and differentiation, and enhance plant growth and plant pathogen defence (Verbon and Liberman, 2016). However, some studies also imply that the presence of root microorganisms accelerates the spread of soil viruses (Macfarlane, 2003). Biotic stress promotes the defensive response of crops, which affects crop growth and yield (Philippot et al., 2013; Liu et al., 2020; Saijo and Loo, 2020). Therefore, the balance between plant and soil microorganisms needs to be focused on (Bennett and Klironomos, 2019).

Eggplant (*Solanum melongena* L.) is widely cultivated in facilities worldwide, and its fruit yield depends on the irrigation amount and soil nutrient availability. However, few studies have been conducted on the effect of the irrigation amount on the eggplant rhizosphere microbial community and rhizosphere soil nutrient supply. In this study, we studied the influence of the irrigation amount on the soil microorganisms, rhizosphere environment, yield and quality of eggplants grown in solar greenhouse with the aim of investigating the effects of different irrigation amounts on the following topics: (1) the eggplant yield, soil characteristics, soil microbial community structure and function; (2) the correlation between microorganisms and soil environmental factors; and (3) the effects of microbial community differences on eggplant yield, with the overarching goal of determining the most suitable irrigation amount for eggplant planting in facilities. Our research will benefit agricultural irrigation and water-saving efforts, improve water use efficiency, and have a positive impact on agricultural production.

## Materials and methods

### Plant material and growth conditions

- Eggplant (*Solanum melongena* L. cv. ‘Blitha’) was used as the model plant for the assays. The basic physical and chemical properties of the tested soil in the solar greenhouse (N: 36°09′35.67′; E: 117°09′33.81′) were as follows: pH 6.94, EC 1.35 mS cm^−1^, and field capacity 38.8%. The available nitrogen, phosphorus and potassium contents were 186.3 mg kg^−1^, 157.0 mg kg^−1^ and 344.0 mg kg^−1^, respectively. Different row spacings were used for planting, with a large row spacing of 80 cm, a small row spacing of 60 cm, and a plant spacing of 50 cm. The area of each test plot was 29.4 m^2^ (L × W = 7 m × 4.2 m), which was replicated three times and arranged randomly. Each treatment was separated by a thick plastic film with a depth of 60 cm to ensure that there was no interference by water exchange among the treated soils. Drip irrigation was adopted, and nutrients and water were supplied by the integrated water and fertilizer solution. The fertilizer supply was N 21.44 kg hm^−2^ 15 days, P_2_O_5_ 19.29 kg hm^−2^ 15 days, and K_2_O 56.81 kg hm^−2^ 15 days in the seedling stage and N 41.45 kg hm^−2^ 15 days, P_2_O_5_ 34.30 kg hm^−2^ 15 days, and K_2_O 86.83 kg hm^−2^ 15 days in the flowering and fruit setting stage, full fruiting and fruit setting stage and harvesting stage.
- Eggplants in the autumn–winter crop were planted on September 8, 2019, and harvested on January 15, 2020. The eggplant growth period was divided into 4 growth stages: the seedling stage (September 18–October 17, 2019), the flowering and fruit setting stage (October 18–November 16, 2019), the full fruiting stage (November 17–December 16, 2019) and the harvesting stage (December 17 2019–January 15, 2020). In this study, the maximum water demand during eggplant cultivation was calculated by the water balance method (Yuan et al., 2004), which was 200.48 m^3^ hm^−2^ 15 days^−1^ at the seedling stage, 334.14 m^3^ hm^−2^ 15 days^−1^ at the flowering and fruiting stage, 451.08 m^3^ hm^−2^ 15 days^−1^ at the full fruiting stage, 350.84 m^3^ hm^−2^ 15 days^−1^ at the harvesting stage. The irrigation amount 100% as the control (CK), and the specific irrigation amounts for the T1 (10% reduction), T2 (20% reduction) and T3 (30% reduction) treatments are shown in Table 1. Drip fertigation was used for irrigation. The 15-day irrigation amount was divided into three times, one time every 5 days. Irrigation water and fertilizers were prepared together every 5 days and stored in buckets, irrigated at once.

**Table 1:** The irrigation amounts of different treatments.

	Irrigation amount(m^3^ hm^−2^ 15 d^−1^)			
Treatments	Seedling stage	Flowering and fruit setting stage	Full fruiting stage	Harvesting stage
CK	200.48	334.14	451.08	350.84
T1	180.43	300.6	405.97	315.76
T2	160.38	267.31	360.86	280.67
T3	140.34	233.9	315.76	245.59

### Determination of plant height and leaf area index

After planting, 5 eggplant seedlings were marked with the same growth in each treatment. Plant height was measured from 0.5 cm above the graft union to the growth point. The measurement was performed every 15 days.
The effects of different treatments on leaf area were shown by the leaf area index (LAI; Watson, 1947; Song and Huang, 1987). The formula for LAI is as follows:

$$LAI=\overset{m}{\underset{j=1}{\sum }}\overset{n}{\underset{i=1}{\sum }}\left(L_{ij}\times B_{ij}\times K\right)\frac{\rho }{m}$$

*L_ij_*: Length of the selected leaves (cm).

*B_ij_*: Width of the selected leaves (cm).

*m*: The number of plants.

*n*: Total leaf number of the *i*-th (the tested) eggplant.

*ρ*: The population density of eggplant (plant m^−2^); the *K* value is 0.7 (our previous pre-experiments examined K values in leaf area index calculations for different varieties of eggplant by leaf area meter (CI-202, CID Bio-science, US) and leaf length and width calculation method, respectively. The results showed that the coefficient values (*K*) between the leaf area data measured by the leaf area meter and the calculated by length and width were from 0.66 to 0.71. For convenience of calculation, we used *K* = 0.7 in this study).

### Determination of photosynthetic characteristics

Photosynthetic characteristic data were detected in the full fruiting stage.

The photosynthetic pigments were measured using the following method: 0.2 g of the leaves (without veins) was weighed into a 30 ml test tube, 20 ml of a 1:1 (V:V) mixture of absolute ethanol and acetone was added, and the mixture was sealed and stored in the dark for 48 h until the leaves were white and visible under ultraviolet light. The optical density (OD) values were measured at 663 nm, 646 nm and 470 nm in a spectrophotometer, and the chlorophyll and carotenoid contents were calculated according to the method of Zivcak et al. (2014). This process was repeated 3 times, and the average value was taken.

The photosynthetic parameters were measured using the following method: on a sunny day from 9:00 to 11:30, the third flat functional leaf was selected from the top, and the net photosynthetic rate (*P*n), stomatal conductivity (*Gs*), intercellular CO_2_ concentration (*Ci*), and transpiration rate (*Tr*) were measured with the LI-6400/XT portable photosynthesis instrument produced by Li-cor (United States). A built-in light source with a light intensity of 500 μmol m^−2^ s^−1^ was used, the temperature was 27 (±1) °C, and the air CO_2_ concentration was 420 μmol mol^−1^. For each treatment, 5 plants were randomly selected, and the average value was calculated.

### Determination of root system development index

Root length, root surface area, and root volume were measured by a MICROTEK root analysis system (Shanghai Zhongjing Co. Ltd., China). The data were analysed by LD-WinRHIZO software. Root vigour was measured using the following method: 0.5 g of the root was mixed with 10 ml of a 1:1 mixed 0.4% 2,3,5-triphenyl-2H-tetrazolium chloride (TTC) solution and 0.067 mol L^−1^ pH 7 phosphate buffer and kept in the dark at 37°C for 4 h, and 2 ml 1 mol L^−1^ sulfuric acid was added to stop the reaction. The sample was ground with 4 ml ethyl acetate and a small amount of quartz sand. The extract was transferred to a test tube, washed with ethyl acetate and diluted to 10 ml. A blank was used as a control, and the OD_485_ value was determined (Zhao et al., 2002).

### Soil physicochemical characteristics and enzyme activities

Soil samples were collected by removing the plant roots during the harvesting stage. The soil sample was air-dried naturally, ground and passed through a 60-mesh sieve to determine the nutrient content. The available nitrogen content was determined by the alkaline solution diffusion method, the available phosphorus content was determined by the sodium bicarbonate method, and the available potassium content was determined by the ammonium acetate extraction-flame photometer method (Bhandari et al., 2002).

The colorimetric method was used to detect sucrase, urease, and alkaline phosphatase activity (Tabatabai and Bremner, 1969; Wyszkowska and Wyszkowski, 2010). The titration method was used to detect catalase activity (Zaborowska et al., 2019).

For catalase activity, 2 g of air-dried soil was placed in a 100 ml conical flask with 40 ml of distilled water and 5 ml of 0.3% hydrogen peroxide solution and shaken for 20 min, and then 1.5 mol-L^−1^ H_2_SO_4_ solution was added to stabilize the undecomposed hydrogen peroxide. The solution was filtered, and 25 ml of the filtrate was titrated with 0.1 mol ml^−1^ KMnO_4_ to the light red endpoint. The hydrogen peroxidase (catalase) activity was expressed as the number of millilitres of 0.1 mol ml^−1^ KMnO_4_ in 1 g of soil after 20 min.

For sucrose activity, 5 g of air-dried soil was placed in a 50 ml conical flask with 15 ml of 8% sucrose solution, 5 ml of phosphate buffer (pH 5.5), and 5 ml of toluene, shaken well and put in a constant-temperature chamber at 37°C for 24 h. After filtering, 1 ml of filtrate was mixed with 3 ml of 3,5-dinitrosalicylic acid, placed in a boiling water bath for 5 min, and cooled under running water for 3 min; then, the volume was adjusted to 50 ml, and the absorbance at 508 nm was measured. The 24 h 1 mg of glucose per g soil reflects the sucrase activity.

For urease activity, 5 g of air-dried soil was placed in a 50 ml conical flask with 1 ml of toluene, 10 ml of 10% urea solution and 20 ml of citrate buffer (pH 6.7), shaken well and placed in a constant-temperature chamber at 37°C for 24 h. After filtering, 3 ml of filtrate was diluted to 20 ml with distilled water and then mixed with 4 ml of sodium phenol and 3 ml of sodium hypochlorite solution, shaken well, and incubated for 20 min. The volume was adjusted to 50 ml, and the absorbance at 578 nm was measured. The milligrams of NH_3_-N in 1 g of soil after 24 h reflected the urease activity.

For alkaline phosphatase activity, 5 g of air-dried soil was placed in a 50 ml conical flask with 5 ml of toluene and 20 ml of 0.5% sodium benzene phosphate solution, shaken well, and then placed in a constant-temperature chamber at 37°C for 2 h. After filtering, 5 ml of filtrate was mixed with 20 ml of distilled water, 0.25 ml of ammonium chloride-ammonium hydroxide buffer (pH 9.8), 0.5 ml of 4-amino antipyrine solution, and 0.5 ml potassium ferricyanide solution, shaken well, and then adjusted to 50 ml. The absorbance was measured at 510 nm within 15 min, and the alkaline phosphatase activity was calculated as milligrams of P_2_O_5_ in 100 g of soil after 2 h.

### Fruit quality determination

Eggplant fruits that had reached the same level of maturity under different treatments were collected, and the fruit quality indices were measured, including soluble protein, soluble sugar, vitamin C and free amino acid (Gutiérrez-Miceli et al., 2007; Zaller, 2007).

The soluble proteins were detected by the Coomassie Brilliant Blue method. The eggplant flesh was collected, and 1 g was added to 3 ml of phosphate buffer (pH = 7.8, 0.05 mol L^−1^), ground in an ice bath, and centrifuged at 10000 rpm and 4°C for 20 min. Then, 20 μl supernatant was taken and allowed to stand for 2 min, and its absorbance was measured at 595 nm.

The soluble sugars were detected by the enthrone colorimetric method. Eggplant flesh (0.3 g) was placed in a test tube with 10 ml of distilled water, sealed, and placed in a boiling water bath for 30 min to prepare an extract; the extraction of the flesh was repeated twice. The combined extracts were placed in a 25 ml volumetric flask and filtered, and 0.5 ml of filtrate was mixed with 1.5 ml of distilled water (for the blank without filtrate, 2 ml of distilled water was used), 0.5 ml of ethyl anthraquinone acetate reagent, and 5 ml of concentrated sulfuric acid, shaken thoroughly, sealed, placed in a boiling water bath for 1 min, and allowed to cool naturally; then, the OD value at 630 nm was measured.

Vitamin C (Vc) content was determined by the 2,6-dichlorophenol-indophenol titration method. Eggplant flesh (5.0 g) was placed in a mortar with 5 ml of 2% oxalic acid, ground into a homogenate, and transferred to a 50 ml volumetric flask; the volume was adjusted with 2% oxalic acid, and then it was filtered. The filtrate was pipetted into a 50 ml conical flask and titrated with calibrated 0.1% 2,6-dichlorophenol indophenol solution until it was peachy red and did not fade for 15 s. The amount of dye added was recorded.

The free amino acid amount was detected by the ninhydrin colorimetric method. Eggplant flesh (0.5 g) was placed in a mortar, and 5 ml of 10% acetic acid was added and ground into a homogenate. The residue was rinsed with ammonia-free water, adjusted to 50 ml, filtered and prepared for use. Then, 1 ml of filtrate was placed in a 20 ml stoppered test tube, mixed well with 1 ml of ammonia-free distilled water, 3 ml of ninhydrin reagent, and 100 μl of 0.1% ascorbic acid, sealed, placed in a boiling water bath for 15 min, and cooled under running water until the red colour faded to blue–purple. Then, it was adjusted to 20 ml with 60% ethanol, and the absorbance was detected at 570 nm.

### Yield statistics

After planting, each plot was marked with 20 plants for yield statistics. The eggplant plant was subjected to the double-stem pruning method. The individual mature fruit were weighed, and the yield was calculated per plant and converted into yield per hectare.

### Soil microorganism DNA extraction and sequencing

Bacterial and fungal DNA was extracted using a PowerSoil DNA Isolation Kit (MoBio, United States). Common primers were used to amplify 338F (5′-ACTCCTAGGGAGCA-3′) and 806R (5′-GGACTCHVGGGTWTTAT-3′) of the V3-V4 region of the bacterial 16S rRNA gene binding adapters and barcode sequences (Quast et al., 2013). Fungal ITS1 primers were used for ITS1 (5′-CTGTCATTAGGGAGAGA-3′), and ITS2 (5′-GCTGCGTTCTTCATCGATGA-3′) was amplified by binding adapter sequences and barcode sequences (Kõljalg et al., 2013). The final products were purified, quantified and homogenized to generate a sequencing library. The constructed libraries for sequencing fragment DNA were quality checked and mass concentration was measured with Qubit fluorometer. Library fragment size was measured with Agilent 2,100., and the qualified libraries were sequenced using Illumina HiSeq 2,500 (Illumina, USA). All raw sequences have been deposited in the NCBI sequence read archive under BioProject accession number PRJNA771427. Each treatment had 6 repeats.

### Bioinformatics analysis

Overlapping reads of the samples were spliced using FLASH software (version 1.2.111), and the spliced sequences obtained were the raw label data. The original tags obtained from the splicing were filtered using Trimmomatic software (version 0.33) to obtain high-quality tag data. Uchime software (version 8.1) was used to identify and remove the chimeric sequences to obtain the final data. The tags were clustered using Uparse software (version 7.0.1090) to obtain operational taxonomic units (OTUs) with 97% similarity. The tags were based on bacterial 16S sequences from the SILVA database (Release 138) and fungal ITS sequences from the UNITE database (Release 8.0). The RDP classifier (version 2.24) was used to classify the taxa with a minimum reliability estimate of 80%. The alpha diversity of the microbial communities was analysed by Mothur (version v.1.305) using Chao1 and ACE richness estimators, and the microbial diversity was measured using Shannon–Wiener and Simpson diversity indices. The FAPROTAX database6 linking species taxonomy and functional annotations was constructed and then linked to the OTU taxonomic table and the FAPROTAX database to predict the function of the bacterial communities (Louca et al., 2016). Fungal gene sequence information was obtained from Funguild7 associated with fungal function and used to predict fungal community function (Xie et al., 2020). The relative abundance of the species in different samples was determined using STAMP8 with the G-test and Fisher’s test. A two-sample t test was used to determine significant differences with a *p* value threshold of 0.05 (Chen et al., 2021).

### Data analysis and statistics

The bioinformatics data were analysed on the Majorbio Cloud Platform^1^ (Shanghai Majorbio Biopharm Technology Co., Ltd.). The data were analysed and plotted (Venn diagram, community bar chart) using R Programming Language (version 3.3.1). Circos was plotted using Circos-0.67-7 software, and significant differences were tested using analysis of variance and the least significant difference test of the agricolae package (version 1.3.3). Linear discriminant analysis (LDA) was performed on six samples based on the taxonomic composition conditioned from the phylum to genus level using LEfSe software to identify communities or species that produced significant differential effects with a log LDA score threshold of 2.0 (Zhou et al., 2019) and to identify the most likely traits to explain the differences between categories (Segata et al., 2011). Principal coordinate analysis (PCoA) based on unweighted UniFrac distance was constructed using R Programming Language (version 3.3.1) to measure the microbial community beta diversity and compare the pairwise differences based on the Wilcoxon rank-sum test. One-way ANOVA was used to test for significant differences between groups, and the stats package of R Programming Language (version 3.3.1) and the SciPy package of Python were used for analysis and plotting. Phylogenetic evolutionary trees were constructed using FastTree software with neighbor-joining at the genus level by Jukes-Cantor distance matrix based on the sequencing, and evolutionary trees were plotted using the R programming language (version 3.3.1). Bacterial metabolic function was predicted, analysed and plotted using the PICRUSt software package. Fungal metabolic function was predicted, analysed and plotted using the FUNGuild package. Prokaryotic (bacterial) ecological functions were predicted using the FAPROTAX database and analysed and plotted using Python. RDA/CCA was based on the experimentally obtained average soil enzyme activity data using R programming language’s vegan package. Correlation heatmaps were analysed and plotted using the R programming language (version 3.3.1; heatmap package). A structural equation model (SEM) of the direct and indirect effects of treatments, physicochemical factors and microorganisms on yield was developed using AMOS software (IBM SPSS Amos 23) by maximum likelihood estimation (MLE). Data from the different treatments were used to construct the SEM. The treatments represent the proportion of organic fertilizer for each treatment: 1, 0.9, 0.8 and 0.7 for the CK, T1, T2 and T3 treatments, respectively. Fungi (Chao1), soil enzymes (catalase, sucrase, urease, and alkaline phosphatase activity) and remaining soil nutrients after harvest (available N, P, and K) represent the RDA1 data for the first axis of the corresponding indicator RDA/CCA. Yields are the sum of the autumn-winter greenhouse eggplant yields.

Statistical analysis was conducted using SPSS 19.0. The figures and table were drawn using GraphPad Prism 7 and Microsoft Excel. All data were analysed by Tukey’s HSD test at significance levels of *p* < 0.05, 0.01 or 0.001.

## Results

### The effect of different irrigation amounts on eggplant plant height, leaf area index, root morphological indices and vitality

**Figure 1:**
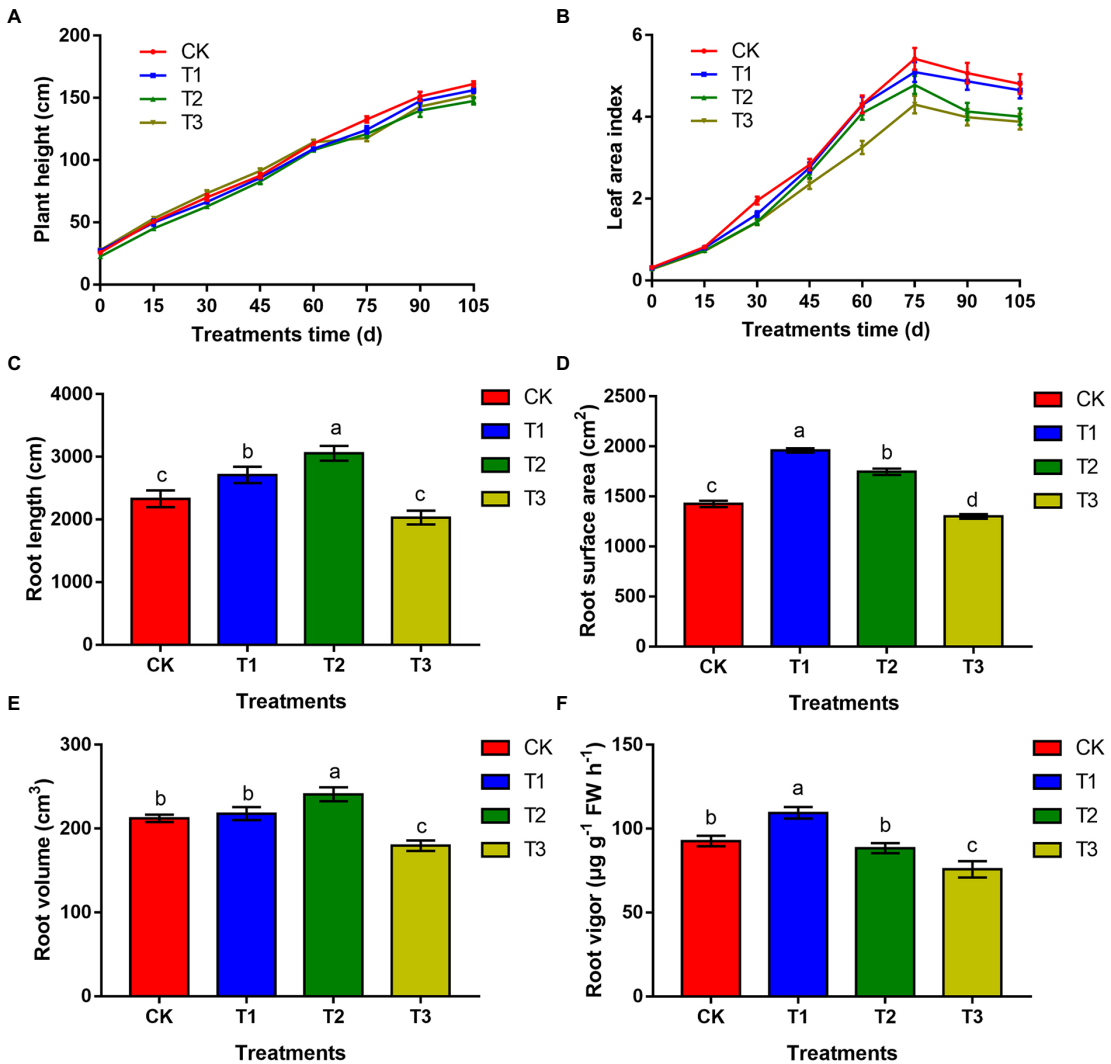
The effects of different irrigation amounts on plant height **(A)**, leaf area index **(B)**, root length **(C)**, root area **(D)**, root volume **(E)**, and root vigour **(F)**. *n* ≥ 3; different letters indicate significant differences (*p* < 0.05).

Eggplant height increased with prolongation of the growth period (Figure 1A). Among all treatments, plants were taller under CK treatment after 60 days, while there was no significant difference between T1 and CK (*p* > 0.05). The leaf area index of eggplant first increased and then decreased (Figure 1B), reaching its maximum at 75 days after planting. Compared with CK, the leaf area index under all treatments decreased. At 75 days after planting, the leaf area index of T1, T2 and T3 decreased by 5.9, 11.8 and 19.7% compared with that of CK, respectively. There was no significant difference (*p* > 0.05) between T1 and CK. At 105 days, the leaf area index of T1, T2 and T3 decreased by 3.3, 16.6 and 19.3% compared with that of CK, respectively, and there was no significant difference between T1 and CK (*p* > 0.05).

The root length of eggplant under T1 and T2 increased significantly, by 16.3 and 31.1%, respectively, compared with that of CK. Under T3, it was significantly reduced by 12.9% compared that of CK (Figure 1C). The root surface area increased significantly by 37.5 and 22.6% under T1 and T2, respectively, compared with that of CK, and the root surface area under T3 was significantly lower than that of CK (Figure 1D). The root volume was the largest under T2, with a significant increase of 13.6% compared with that of CK (*p* < 0.05). There was no significant difference between T1 and CK (*p* > 0.05), but the root volume was significantly reduced in T3 significantly, by 15.3% (Figure 1E). The root vigour was highest under T1, which was 18.7% higher than that under CK, and the difference was significant (*p* < 0.05). The root vigour under T2 was 5.3% lower than that under CK, but the difference was not significant (*p* > 0.05). The root vigour under T3 was significantly lower than that under CK (Figure 1F).

### The effect of different irrigation amounts on photosynthetic parameters in the leaves of eggplants

The chlorophyll a content was higher under T1 and T3, increased significantly by 15.3 and 14.4% compared to CK (Table 2). There was no significant difference in chlorophyll b content between all treatments and CK (*p* > 0.05). The carotenoid content was highest under T3, 23.3% higher than CK, and the difference was significant, but there was no significant difference between T1, T2 and CK (*p* > 0.05). The chlorophyll (a + b) content of T1 and T3 was significantly higher (9.1 and 9.9%, respectively) than that of CK.

**Table 2:** Effects of different irrigation amounts on photosynthetic pigments content in leaves of eggplants.

Treatments	Chl a content (mg g^−1^ FW)	Chl b content (mg g^−1^ FW)	Carotenoid content (mg g^−1^ FW)	Chl a/Chl b	Chl a + Chl b (mg g^−1^ FW)
CK	2.15 ± 0.14b	0.79 ± 0.13a	0.73 ± 0.01b	2.79 ± 0.57a	2.94 ± 0.07b
T1	2.48 ± 0.08a	0.72 ± 0.05a	0.77 ± 0.04b	3.45 ± 0.20a	3.21 ± 0.11a
T2	2.33 ± 0.08ab	0.73 ± 0.08a	0.70 ± 0.03b	3.21 ± 0.43a	3.06 ± 0.02ab
T3	2.46 ± 0.05a	0.77 ± 0.07a	0.90 ± 0.02a	3.20 ± 0.06a	3.23 ± 0.07a

*n* = 3, different letters indicate significant differences (*p* < 0.05).

The net photosynthetic rate (*Pn*) in leaves of eggplant was significantly higher under T1 and T2, with 33.0 and 26.4% increases in comparison with that under CK, but there was no significant difference between T1 and T2 (*p* > 0.05; Table 3). There was no significant difference in the transpiration rate (*Tr*) between T1 and T2 and CK (*p* > 0.05), but the Tr was significantly lower, by 13.8%, under T3 than under CK. Compared with CK, stomatal conductance (*Gs*) was significantly highest under T1, with a value 12.1% higher than that under CK. The intercellular carbon dioxide concentration (*Gi*) under T1 was not significantly different from that under CK, while the values under T2 and T3 were significantly lower than that under CK. All treatments significantly improved the water use efficiency (*Pn/Tr*), which was 23.0% higher under T1 than under CK, but there was no significant difference among T1, T2 and T3 (*p* > 0.05).

**Table 3:** Effects of different irrigation amounts on photosynthetic parameters in leaves of eggplants.

Treatments	*Pn* (μmol m^−2^ s^−1^)	*Tr* (mmol m^−2^ s^−1^)	*Gs* (mmol m^−2^ s^−1^)	*Ci* (μmol mol^−1^)	*Pn/Tr* (μmol mmol^−1^)
CK	9.57 ± 0.19b	15.77 ± 0.67a	607.67 ± 14.26b	416.00 ± 9.34a	0.61 ± 0.02b
T1	12.73 ± 0.36a	16.97 ± 0.36a	681.33 ± 13.75a	407.33 ± 5.16a	0.75 ± 0.01a
T2	12.10 ± 0.24a	16.27 ± 0.61a	637.00 ± 14.31b	373.67 ± 6.95b	0.74 ± 0.01a
T3	10.00 ± 0.54b	13.60 ± 0.39b	544.33 ± 11.81c	381.33 ± 3.61b	0.74 ± 0.03a

*n* = 3, different letters indicate significant differences (*P* < 0.05).

### The effect of different irrigation amounts on soil enzyme activity and the contents of available nitrogen, phosphorus, and potassium

**Figure 2:**
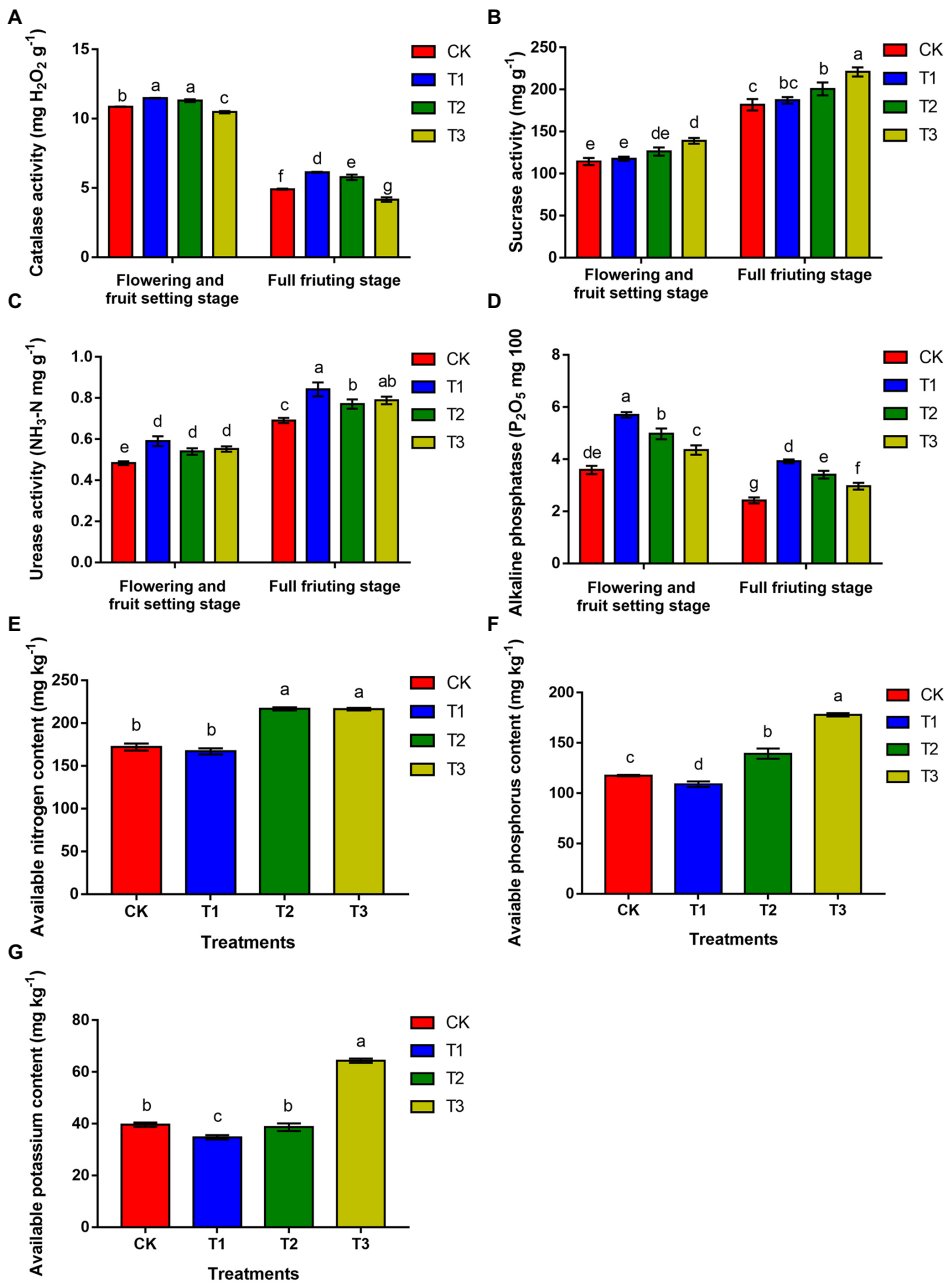
The effects of different irrigation amount on catalase activity (CAT; **A**), sucrase activity (SA; **B**), urease activity (UA; **C**), alkaline phosphatase (APA; **D**), available nitrogen content **(E)**, available phosphorus content **(F)** and available potassium content **(G)**. *n* ≥ 3; different letters indicate significant differences (*p* < 0.05).

As shown in Figure 2, during the flowering and fruit setting stage, soil catalase activity was 5.8% higher under T1 than under CK, and the difference was significant, but there was no significant difference between T1 and T2 (*p* > 0.05). Soil alkaline phosphatase activity and urease activity were highest under T1, with values that were 59.2 and 21.7% higher than those under CK, respectively, and the differences were significant. Soil sucrase activity was significantly higher under T3, and there was no significant difference between the T1 and T2 treatments and CK (*p* > 0.05). At the full fruiting stage, compared with CK, soil catalase and alkaline phosphatase activities were 25.1 and 62.0% higher under T1, respectively, and the difference was significant. The urease activity was significantly higher in T1, with a value 22.9% higher than that in CK, and the urease activity in T2 and T3 was also higher than that in CK. The sucrase activity increased with decreasing irrigation amounts and was highest under T3, in which it was 21.5% higher than that under CK, a significant difference. There was no significant difference between T1 and CK (*p* > 0.05).

The results showed that the contents of available nitrogen (Figure 2E), phosphorus (Figure 2F) and potassium (Figure 2G) were higher under T3 than under CK by 25.9, 51.4 and 62.5%, respectively. T2 followed, but there was no significant difference in available nitrogen content between T2 and T3 or between T1 and CK (*p* > 0.05). However, the available phosphorus content in T1 was significantly lower than that in CK (*p* < 0.05). The available potassium content was also reduced by 12.3% in T1 compared to that in CK, and the difference was significant (*p* < 0.05). Above all, these results implied that the rhizosphere soil nutrient content was reduced with increasing irrigation amounts, which may be due to the high nutrient utilization rate.

### Effects of different irrigation amounts on the OTU number and diversity of soil microbial communities

All samples were sequenced, the same number of obtained OTUs were extracted randomly and annotated by species taxonomy, and the corresponding abundance information of each OTU annotation result in each sample was counted and displayed as a rank–abundance curve (Supplementary Figures S1A,D). The pan/core species curve was relatively flat, indicating that the sequencing sample size was sufficient (Supplementary Figures S1B,C,E,F).

**Figure 3:**
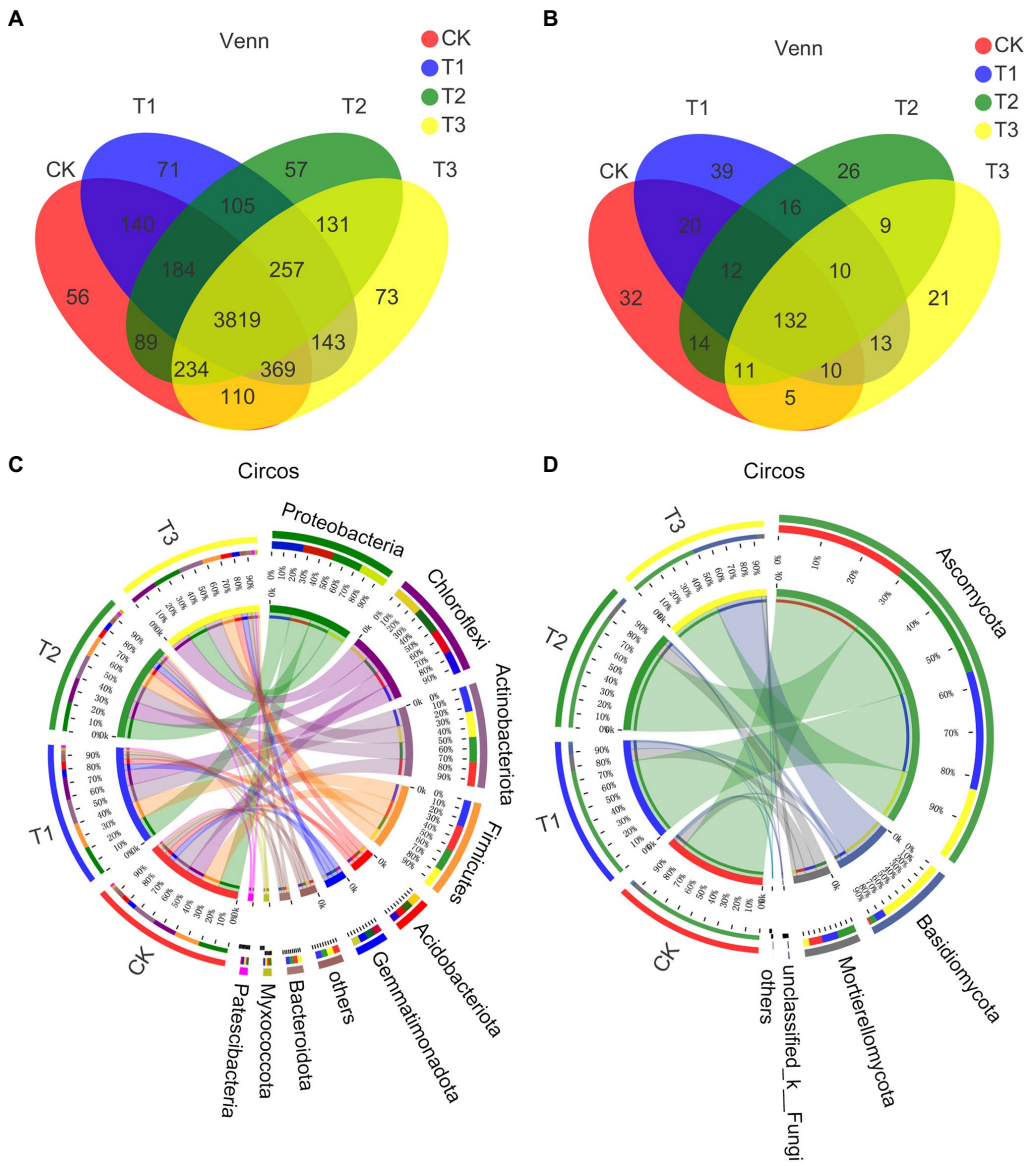
The effect of different irrigation amounts on the similarity and overlap of bacterial **(A)** and fungal **(B)** OTU compositions in soil; the similarity level was higher than 97% (Venn). Circos reflects the distribution ratio of dominant phyla in different treatments and the distribution ratio of each dominant phylum in different treatments, including bacteria **(C)** and fungi **(D)**. *n* = 6.

Table 4 shows that the bacterial OTU numbers were higher under T3 and lower under T2; the fungal OTU numbers were lower under T3 and higher under T1, but there were no significant differences among the treatments (*p* > 0.05). A total of 3819 bacterial OTUs were present under all treatments, with approximately 74.3–78.3% of OTUs per treatment, and approximately 1.0–1.5% of the OTUs per treatment were different from the other treatments (Figure 3A). A total of 132 fungal OTUs were present under all treatments, accounting for 52.4–62.6% of OTUs per treatment, and approximately 10–17% of the OTUs per treatment were different from those found in the other treatments (Figure 3B). Thus, compared to bacteria, the fungal OTUs differed more between each treatment.

**Table 4:** Effects of different irrigation amounts on alpha diversity of bacteria and fungi in OTU level.

Microbial species	Treat-ment	ACE index	Chao1 index	Simpson index	Shannon index	OTU number
Bacteria	CK	3961.48 ± 96.06a	3960.80 ± 89.84ab	0.00304 ± 0.00024a	6.70 ± 0.04a	5,001 ± 105a
	T1	4016.27 ± 316.62a	3981.07 ± 324.36b	0.00326 ± 0.00091a	6.69 ± 0.20a	5,088 ± 274a
	T2	3893.9 ± 59.74a	3900.04 ± 57.41ab	0.00289 ± 0.00020a	6.71 ± 0.05a	4,876 ± 84a
	T3	4072.4 ± 282.30a	4034.31 ± 295.99a	0.00268 ± 0.00028a	6.78 ± 0.10a	5,136 ± 278a
Fungi	CK	228.82 ± 30.44a	229.72 ± 29.07ab	0.151 ± 0.068b	2.74 ± 0.46a	236 ± 25a
	T1	275.52 ± 38.89a	279.98 ± 36.36a	0.117 ± 0.046b	2.92 ± 0.44a	252 ± 38a
	T2	244.32 ± 28.12a	250.86 ± 30.74ab	0.139 ± 0.052b	2.64 ± 0.30ab	230 ± 23a
	T3	229.14 ± 30.49a	226.38 ± 34.48b	0.255 ± 0.067a	2.07 ± 0.33b	211 ± 35a

*n* = 6, different letters indicate significant differences (*P* < 0.05).

The results obtained from sequencing were used to analyse all OTUs based on the OTU level, and the same number of obtained OTUs was randomly extracted to analyse the soil microbial community. The flatness of the rarefaction curve indicates that the amount of sequencing data is reasonable (Supplementary Figure S2). The results showed that the alpha diversity of the soil microbial community was affected differently by different irrigation amounts (Table 4), with the fungal ACE index being higher under T1 and significantly lower under CK and T3. The bacterial Chao1 index was higher under T3 and significantly lower under T1, while the fungal Chao1 index was higher under T1 and significantly lower under CK and T3. These results indicated that the fungal community richness was higher under T1 and that the bacterial community richness was higher under T3. The fungal Simpson index was highest under T3 and significantly lower under the other treatments. The fungal Shannon index was higher under T1, but there was no significant difference (*p* > 0.05) between CK, T1 and T2, and it was significantly lower under T3. This finding indicates that the fungal community diversity was highest in T1. The rest of the indices were not significantly different.

**Figure 4:**
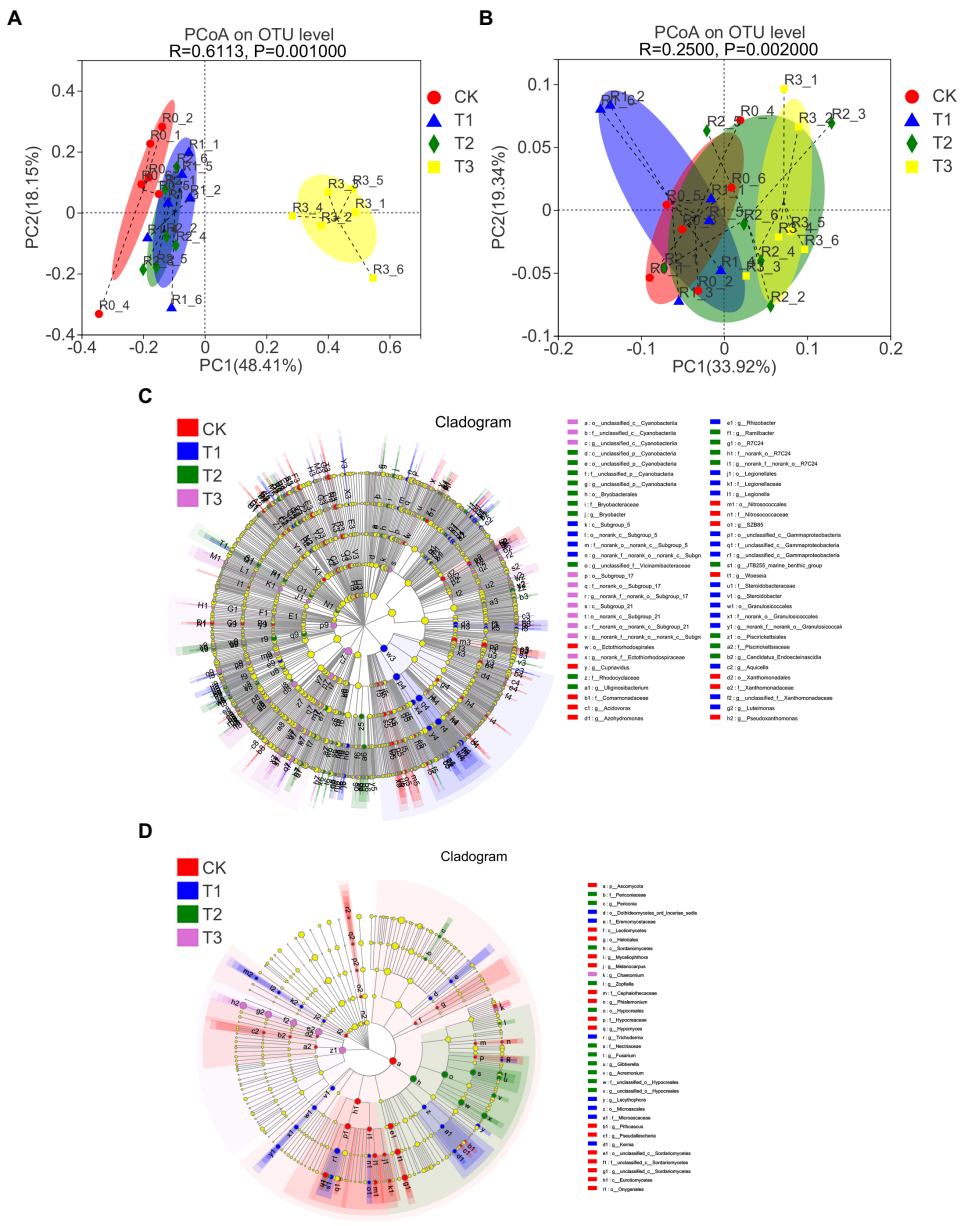
Principal coordinate analysis (PCoA) of bacterial **(A)** and fungal **(B)** OTU levels in soil at different irrigation amounts with an unweighted UniFrac distance algorithm. LEfSe analysis from the phylum to genus level, including bacteria **(C)** and fungi **(D)**, by the nonparametric factorial Kruskal–Wallis (KW) sum-rank test. *n* = 6.

The beta-diversity of soil microbial communities was also affected differently by different irrigation levels. PCoA based on the weighted UniFrac distance algorithm showed that the fungal communities were more dispersed than the bacterial communities under different irrigation treatments, where the fungal communities of T3 were significantly dispersed from those of the other treatments, the fungal communities under T1 and T2 were significantly dispersed from that of CK, and the bacterial communities under T3 were significantly separated from those of CK (Figures 4A,B; Supplementary Figures S3A,B). The results of ANOSIM/Adonis analysis showed that the between-group differences were significantly greater than the within-group differences for all treatments (Supplementary Figures S3C,D).

### Effects of different irrigation amounts on the structure of soil microbial communities

The community distributions of bacteria and fungi are shown in the Circos and community bar diagrams with relative abundance, including differences at the bacterial phylum level (Figure 3C; Supplementary Figure S4A) and at the fungal phylum level (Figure 3D; Supplementary Figure S4B) and genus level (Supplementary Figures S4C,D). Ten bacterial phyla were detected: Proteobacteria, Chloroflexi, Actinobacteriota, Firmicutes, Acidobacteriota, Gemmatimonadota, Bacteroidota, Myxococcota, Patescibacteria and others. Proteobacteria was found to be the most abundant phylum under each treatment except T3, with 21.86, 22.33, 21.61 and 20.19% in CK, T1, T2 and T3, respectively. A higher relative abundance of Chloroflexi was found under T3 (20.54%). Chloroflexi and Acidobacteriota had lower relative abundances under T1, and their relative abundances increased gradually with decreasing irrigation amount. Five fungal phyla were detected, and the relative abundance of Ascomycota was higher under each treatment, with values of 86.91, 79.06, 81.67 and 48.62% under CK, T1, T2 and T3, respectively, with the value under T3 representing a significantly lower value. A total of 17 fungal genera were detected, with a higher relative abundance of *Cladosporium* under all treatments except T3, and a higher relative abundance of *Wallemia* was detected in T3.

While the significant differences between species among treatments are shown in Supplementary Figure S5, the differences were significant for Deinococcota at the bacterial phylum level (*p* < 0.01) and for the rest of the phyla, as shown in the Supplementary Figure S5A. The difference between Ascomycota and Basidiomycota at the level of the fungal phylum was significant (*p* < 0.001), while the differences in rest of the phyla in Supplementary Figure S5B were significantly different (*p* < 0.05). Significant differences were found between *Norank A4b* and *Norank SBR1031* (Chloroflexi; *p* < 0.001), *Sphingomonas*, *Norank Saccharimonadales* and *Truepera* (*p* < 0.01) at the level of the bacterial genus, and the rest were significantly different (*p* < 0.05; Supplementary Figure S5C). Significant differences were found for *Wallemia* (*p* < 0.001), *Fusarium*, *Phialosimplex* and *Lecythophora* (*p* < 0.01) at the level of fungal genera, and the rest of the genera in the figure (*p* < 0.05; Supplementary Figure S5D).

The LEfSe results showed that there were 60 bacterial communities and 35 fungal communities with significant differences (Figures 4C,D). The bacteria family Sphingomonada ceae and the fungus phylum Ascomycota were the most abundant under CK, the bacteria class Bacilli and the fungus family Aspergillaceae were the most abundant under T1, the bacteria genus norank f A4b and fungus class Sordariomycetes the most abundant under T2, and the bacteria phylum Chloroflexi and fungus class Wallemiomycetes were the most abundant under T3 (SM 2 and 3). Phylogenetic tree-and genus-level reads showed the same results (Supplementary Figure S6).

### Effects of different irrigation amounts on bacterial functional metabolism and fungal trophic patterns

**Figure 5:**
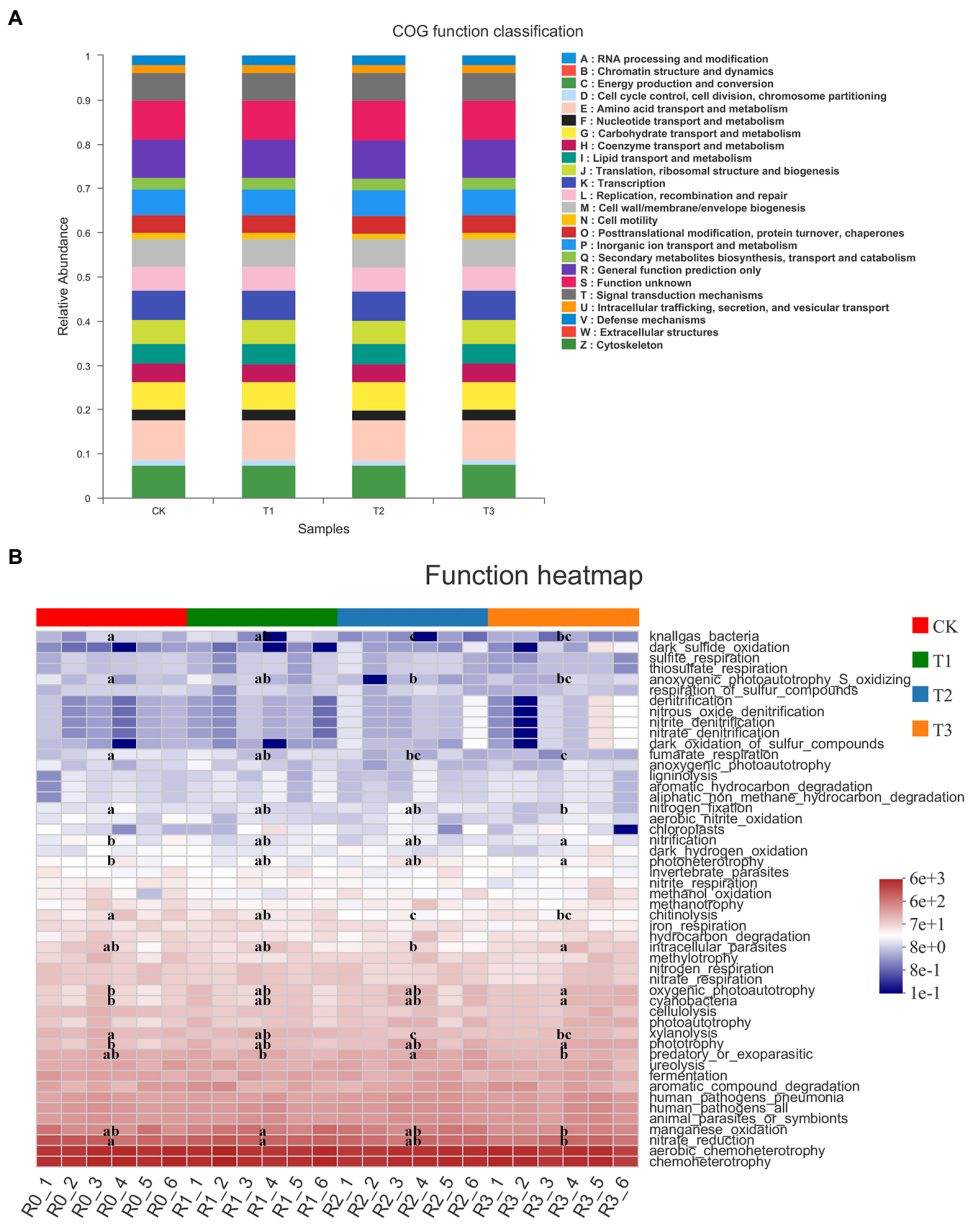
Clusters of Orthologous Groups (COG) functional classification of PICRUSt in bacteria **(A)** of different treatments. The functional heatmap predicated by FAPROTAX in bacteria **(B)** under different treatments. *n* = 6; different letters indicate significant differences (*p* < 0.05).

The bacterial community showed 25 COG functional classifications under all treatments, as predicted by PICRUSt1, with the highest abundance of amino acid transport and metabolism functional classes. However, the differences in each functional class under CK and the three treatments were not significant (Figure 5A). By FAPROTAX annotation, the bacterial community showed 50 functions, among which the relative abundance of chemoheterotrophy and aerobic chemoheterotrophy was higher. The functions with significant differences (*p* < 0.05) in abundance between treatments are shown in Figure 5. Interestingly, these differences were mainly associated with N cycling and nutrient catabolism. With decreasing irrigation amount, nitrate reduction, nitrogen fixation, manganese oxidation, fumarate respiration, anoxygenic photoautotrophy S oxidation, xylanolysis, and chitinolysis were reduced or significantly reduced, but there was no significant difference between T1 and CK (*p* > 0.05). With the decrease in irrigation water, the functions of phototrophy, oxygenic photoautotrophy, photoheterotrophy and nitrification increased or significantly increased (Figure 5B).

**Figure 6:**
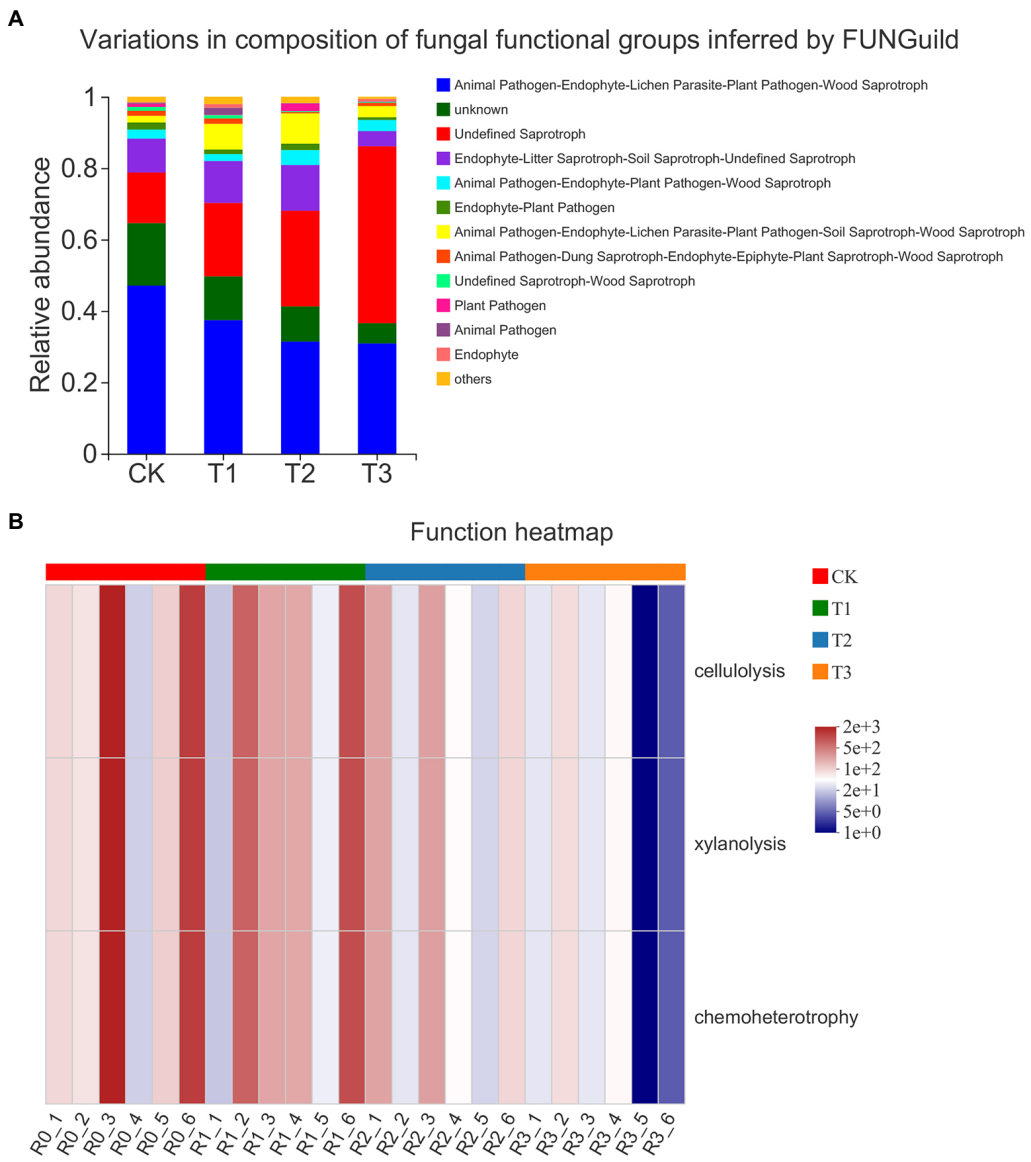
Taxonomic analysis of fungal communities by microecological guild (FUNGuild; **A**) of different treatments. The functional heatmap predicated by FAPROTAX in fungi **(B)** under different treatments. *n* = 6.

The trophic pattern of the fungal community was dominated by saprotrophs (Figure 6A). The proportion of animal endosymbiont–animal pathogen–endophyte–plant pathogen–undefined saprotrophs decreased, and the proportion of undefined saprotrophs increased with decreasing irrigation amount. The proportion of endophyte–litter saprotroph–soil saprotroph-undefined saprotrophs and animal pathogen–endophyte–lichen parasite–plant pathogen–soil saprotroph–wood saprotrophs was higher in T1 and T2 than in CK and T3, while the proportion of animal pathogens was significantly higher under T1, and the proportion of plant pathogens was significantly higher under T2 than under the other treatments. The FAPROTAX functional prediction heatmap showed that the fungal community functions included cellulolysis, xylanolysis and chemoheterotrophy, and the functional abundance gradually decreased with decreasing irrigation amount, but the difference between treatments was not significant (Figure 6B).

### Relationship between microbial community structure and soil enzyme activity in different treatments

**Figure 7:**
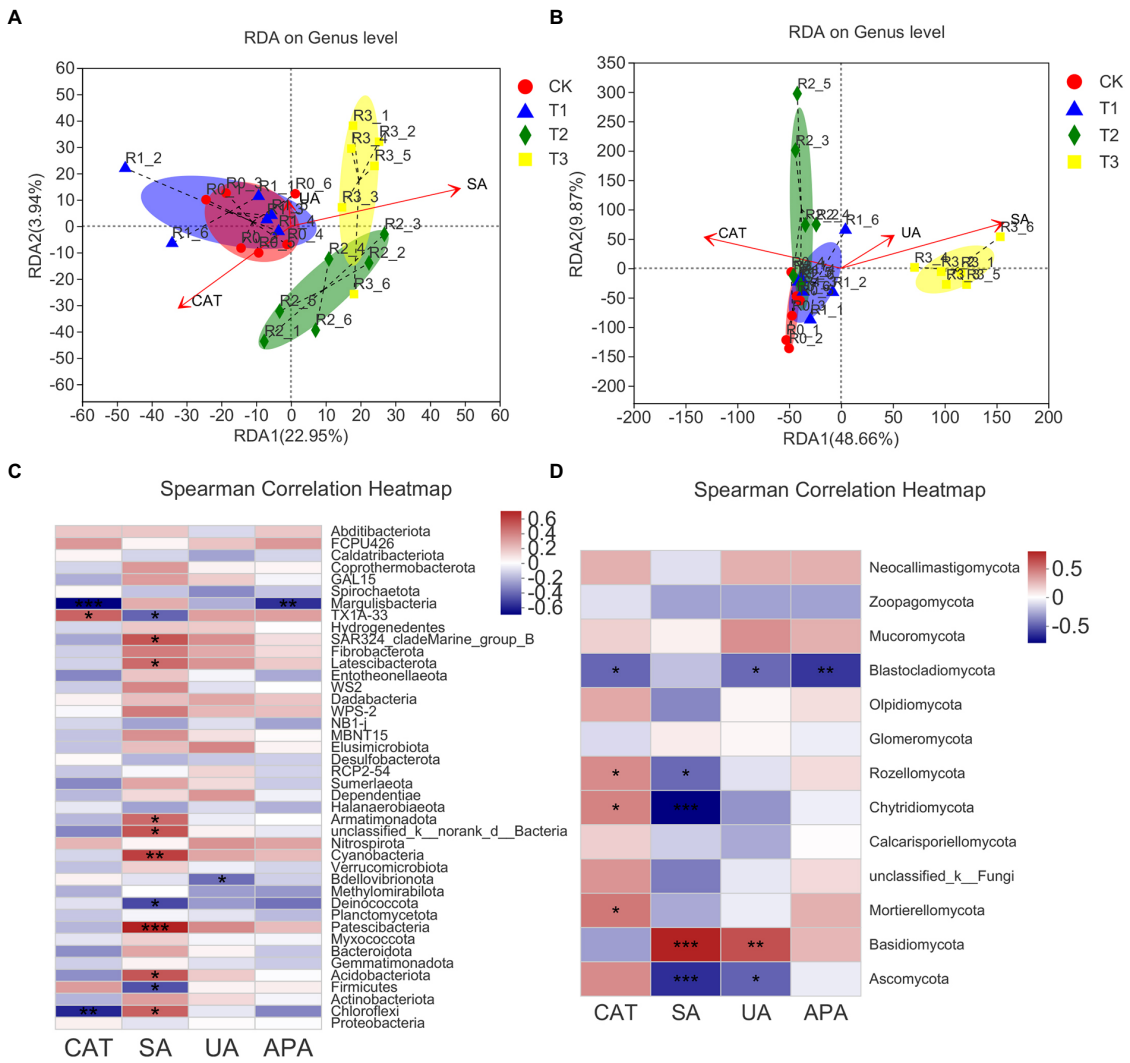
The ranking method developed by redundancy analysis and correspondence analysis (RDA/CCA) in different treatments. The environmental factor adopts the mean value of soil enzyme activity, including bacteria **(A)** and fungi **(B)**, at the genus level. The relationships and correlations between different phyla in bacteria **(C)** and fungi **(D)** with soil enzyme activities. *n* = 6, different stars indicate significant differences (*p < 0.05; **p < 0.01; ***p < 0.001).

The soil microbial community structure was closely related to soil enzyme activity. Overall, the correlation between soil microbial community structure and soil enzyme activity on both dimensions (RDA1 and RDA2) was 26.89% for bacterial communities and 58.53% for fungal communities (Figures 7A,B). Table 5 shows that the soil microbial community structure was significantly correlated (*p* < 0.01) with catalase activity (CAT; bacteria *R* = 0.6251, fungi *R* = 0.7587) and sucrase activities (SA; bacteria *R* = 0.7518, fungi *R* = 0.9080). There was no significant correlation between soil microbial community structure and urease activity (UA) or alkaline phosphatase activity (APA; *p* > 0.05; Table 5).

**Table 5:** Monte Carlo permutation test of soil factors on microbial community structure.

Bacteria	RDA1	RDA2	*r* ^2^	*P*-values	Fungi	RDA1	RDA2	*r* ^2^	*P*-values
CAT	−0.7954	−0.606	0.3908	0.003	CAT	−0.9759	0.2183	0.5756	0.001
SA	0.9835	0.181	0.5652	0.001	SA	0.9752	0.2214	0.8244	0.001
UA	−0.1671	0.9859	0.0142	0.854	UA	0.8786	0.4776	0.1141	0.282
APA	−0.8046	−0.5939	0.044	0.634	APA	−0.4719	0.8816	0.1046	0.304

*n* = 6.

The correlation between the relative abundance of the microbial community at the phylum level and soil enzyme activity under different irrigation treatments was analysed by the Spearman rank correlation coefficient and was shown by a heatmap (Figures 7C,D). Patescibacteria was significantly and positively correlated with SA activity (*p* < 0.001); Cyanobacteria was significantly and positively correlated with SA activity (*p* < 0.01); SAR clade Marine Group B, Latescibacteria, Armatimonadota, unclassified k norank d Bacteria, Acidobacteria, and Chloroflexi were significantly and positively correlated with SA activity (*p* < 0.05); and TX1A-33 was significantly and positively correlated with CAT activity. Margulis bacteria showed a significant negative correlation with CAT and APA activity (*p* < 0.01), and Chloroflexi showed a significant negative correlation with CAT activity (*p* < 0.01). TX1A-33, Deinococcota and Firmicutes showed a significant negative correlation with SA activity (*p* < 0.05), and Bdellovibrionota showed a significant negative correlation with UA activity (*p* < 0.05). Among the fungal communities, Basidiomycota showed a significant positive correlation with SA and UA activity (*p* < 0.001). Rozellomycota, Chytridiomycota, and Mortierellomycota showed a significant positive correlation with CAT activity (*p* < 0.05). Blastocla diomycota showed a significant negative correlation with APA activity (*p* < 0.01) and with CAT activity or UA activity (*p* < 0.05). Ascomycota showed a significant negative correlation with SA activity (*p* < 0.001) and UA activity (*p* < 0.05).

### The effect of different irrigation amounts on the fruit quality and yield of eggplant

As shown in Table 6, the contents of soluble sugars, soluble proteins and free amino acids were all significantly higher under T1 and T2, which were 10.8, 12.3, and 6.7% and 6.6, 6.8, and 3.1% higher than those under CK, respectively. The soluble sugar, soluble protein and free amino acid contents of the fruit under T3 were 4.2, 4.3 and 3.2% higher than those under CK, respectively, values that were not significantly different (*p* > 0.05). There was no significant difference in the vitamin C (Vc) content among all treatments (*p* > 0.05). After yield, compared with CK, T1 increased yield by 3.9%, but the difference was not significant (*p* > 0.05). The yield in T2 and T3 decreased by 14.4 and 16.7%, respectively, compared with that in CK, and the difference between T3 and CK was significant (*p* < 0.05).

**Table 6:** Effects of different irrigation amounts on eggplant fruit quality and yield.

Treatments	Soluble sugar %	Soluble protein (mg g^−1^ FW)	Free amino acid (mg kg^−1^ FW)	Vc (mg kg^−1^ FW)	Yield (kg ha)
CK	3.81 ± 0.09b	2.35 ± 0.10b	499.84 ± 15.33b	55.49 ± 1.05a	111706.5 ± 2038.5b
T1	4.22 ± 0.11a	2.64 ± 0.07a	533.10 ± 11.79a	58.46 ± 1.13a	116001.0 ± 2023.5a
T2	4.06 ± 0.12a	2.51 ± 0.11a	515.37 ± 13.45ab	60.13 ± 0.95a	105586.5 ± 1927.5c
T3	3.97 ± 0.11b	2.45 ± 0.13ab	501.47 ± 10.13b	56.42 ± 1.03a	95571.0 ± 1884.0d

*n* = 3, different letters indicate significant differences (*P* < 0.05).

### Structural equation modelling

**Figure 8:**
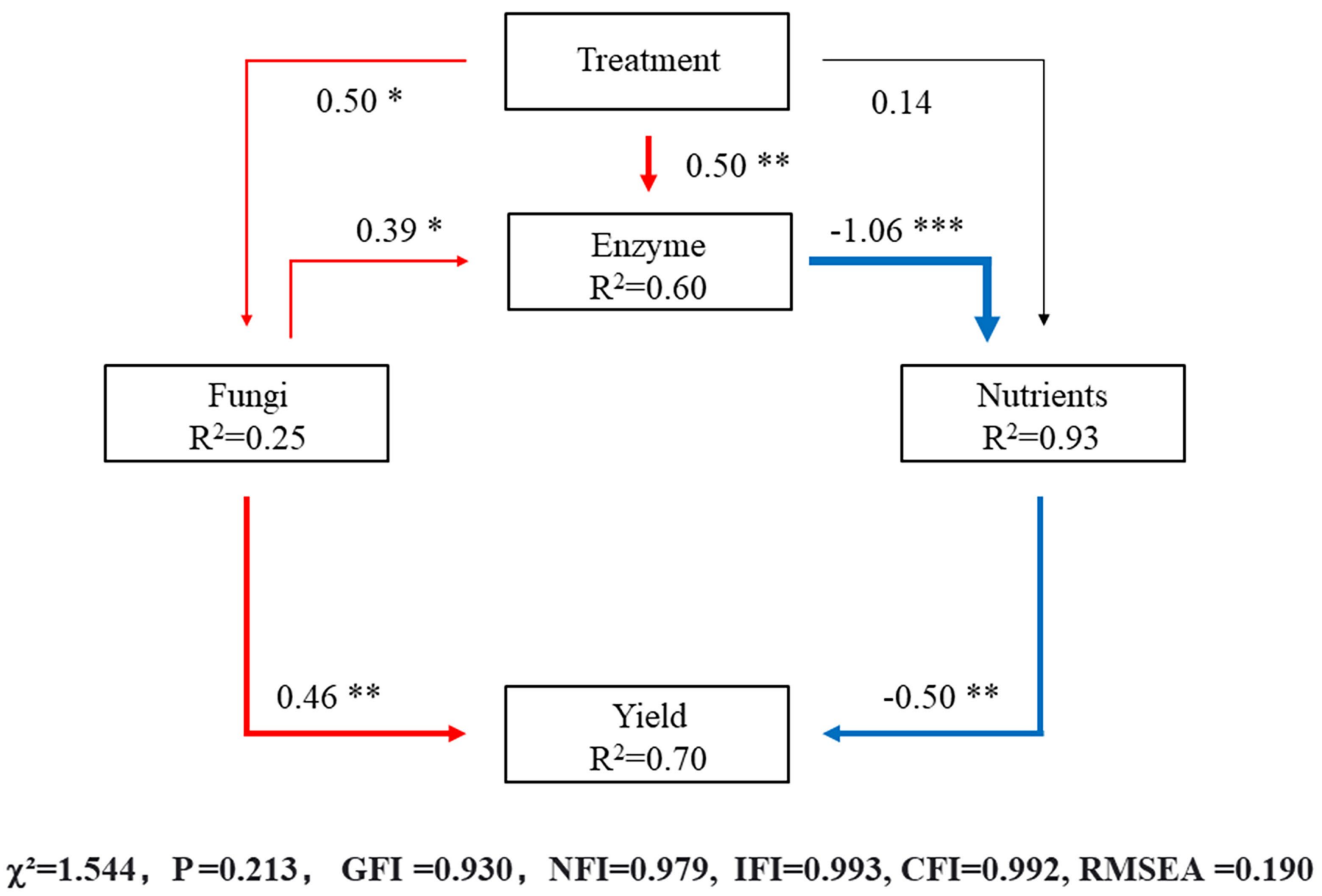
The values above the structural equation model (SEM) lines indicate path coefficients. The red line represents positive path coefficients, the blue line represents negative path coefficients, and the black line represents nonsignificant path coefficients. The width of the arrow indicates the significance of the standard path coefficient (***p < 0.001; **p < 0.01; *p < 0.05).

According to the above results, the effect of different irrigation amounts on the fungal community in the soil was greater than that of bacteria; therefore, the structural equation model (SEM) analysis of the direct and indirect effects of different irrigation levels was based on soil fungi and included soil enzyme activity, soil nutrient residuals and autumn–winter crop yield of eggplants. The model fit the data well (*χ*^2^ = 1.544, *p* = 0.213, Goodness of fit index/GFI = 0.930, Normed fit index/NFI = 0.979, Incremental of fit index/IFI = 0.993, Comparative fit index/CFI = 0.992, Root mean square error of approximation/RMSEA = 0.190) and explained 25% of the variation in soil fungi, 93% of the variation in soil nutrients, 60% of the variation in soil enzyme activity and 70% of the variation in yield of eggplants (Figure 8). The results showed that the different irrigation treatments significantly affected the distribution of the fungal community structure (*λ* = 0.50, *p* < 0.05) and soil enzymes (*λ* = 0.50, *p* < 0.01), but the effect on nutrients was not significant. The fungal community structure had a significant effect on the soil enzymes (*λ* = 0.39, *p* < 0.05). More soil nutrients at the harvesting stage indicated lower nutrient utilization, the effect of soil enzymes on nutrients was extremely significant (*λ* = −1.06, *p* < 0.001), and the yield was significantly affected by the fungal community structure (*λ* = 0.46, *p* < 0.01) and residual soil nutrients (*λ* = −0.50, *p* < 0.01).

## Discussion

As climate change intensifies and the population continues to grow rapidly, water scarcity is becoming increasingly pronounced. This study investigated the effects of irrigation water amounts on eggplant growth and development, yield, rhizosphere microbial community structure and function, and rhizosphere soil physicochemical properties. The results showed that, compared with full irrigation (CK), 10% reduced irrigation (T1) treatment improved plant height, fruit yield and quality as well as photosynthetic rate, transpiration rate, stomatal conductance and water use efficiency. The structure and function of the soil microbial community were also significantly altered. Under the 10% reduced irrigation (T1) treatment, the richness and diversity of the microbial community were significantly increased, the activities of soil catalase, urease and alkaline phosphatase increased, and the N cycling rate and nutrient catabolism also increased.

With the development of the agricultural industry, the demand for technological improvements in the field of agricultural research is growing. Much research focuses on the crop itself in efforts to enhance the potential of the crop (Hwang et al., 2017; Baileyserres et al., 2019), but this focus is far from sufficient. Modern agriculture needs more attention, including crop growth environments, crop growth substrates, agricultural facilities, and root microbes (Rouphael et al., 2018; Saiz-Rubio and Rovira Más, 2020). Water saving is one of the important goals of agricultural production (Chai et al., 2015). Therefore, to achieve the goal of saving water, it is not sufficient to develop agriculture only from the perspective of crops (Zhang et al., 2018). The innovation of agricultural facilities and technologies is also very important (Pretty and Bharucha, 2014).

In this research, we used the calculated theoretical greenhouse eggplant water requirements as a control and reduced the irrigation volume by 10, 20, and 30% relative to the control (100%). The results showed that the treatment in which the irrigation amount was reduced by 10% did not significantly decrease the plant height compared with that in CK, and the difference in leaf area was also not significant. However, after the irrigation amount was reduced by 20% or 30%, the plant height and leaf area decreased, which had an obvious adverse effect on eggplant yield. Therefore, an irrigation amount under the 10% reduced irrigation treatment can ensure the water demands of eggplant during different growth stages. The 10% reduced irrigation treatment promoted the development of the root system and increased the root area and root vigour, thereby increasing the ability of eggplant to absorb nutrients. Meanwhile, the soil enzyme activity and soil available nutrient utilization, including available nitrogen, phosphorus, and potassium, were all improved.

Studies have shown that an ebb-and-flow subirrigation system can be used to increase water use efficiency in greenhouse cucumber planting; the enrichment of specific fungi increases under the nutrient solution, but it does not increase the cucumber disease rate (Dong et al., 2020). Specific microorganisms can promote crop resistance to water loss (Rajabi-Khamseh et al., 2020) but may also affect plant root development (Bennett and Klironomos, 2019). The activities of catalase, urease and alkaline phosphatase were all higher under the 10% reduced irrigation treatment in the flowering and fruit setting and full fruiting stages. However, the available nitrogen, phosphorus and potassium contents in the soil were lower under the 10% reduced irrigation treatment than under the control treatment. These results showed that although the microbial community structure under the 10% reduced irrigation treatment was changed, compared to CK, the vitality and fertility of the soil were significantly improved, and the soil nutrients could be utilized by plants to the greatest extent. An improved root system increases the absorption of soil nutrients by crops and promotes growth (Zhang et al., 2021) through changes in the osmosis capacity of root cells or changes in cell wall characteristics (Al-Yasi et al., 2020). This root-soil interaction can significantly increase the yield and quality of crops (Wang et al., 2020). This impact is multifaceted, including root growth (Luo et al., 2016), phytohormone (Puértolas et al., 2020), soil nutrient cycling (Du et al., 2008), root redox balance regulation (Bogale et al., 2016) and other factors.

The impact of irrigation on crops is multidimensional and can directly affect crop water supply and indirectly affect crop yield and quality by improving soil quality and the soil microbial composition (Rousk et al., 2010; Chodak et al., 2015; Santos et al., 2021). In this study, through the sequencing and analysis of soil microorganisms, it was found that the microbial community structure and function in the soil were changed by decreasing irrigation. Some interesting findings were also found, such as the high abundance of Firmicutes, Basidiomycota, and Mortierellomycota and the low abundance of Acidobacteriota under the 10% reduced irrigation treatment. The genus *Bacillus* of the phylum Firmicutes is considered to be a beneficial microorganism in agroecology, promoting crop growth and enhancing plant immunity (Hashmi et al., 2020). Bacilli (class) are also considered to promote the decomposition of organic fertilizers and enhance the nutrient utilization of rice (Liu et al., 2021). Basidiomycota exhibit the ability to form mycorrhizae in symbiosis with crops and accelerate the decomposition of lignin and cellulose in soil (Vohník et al., 2012; Genre et al., 2020). The abundance of *Wallemia* was significantly increased under T3, and the genus *Wallemia* of the phylum Wallemia is a unique drought-and salt-tolerant fungus (Zalar et al., 2005; Kralj Kunčič et al., 2010), which also suggested that the soil moisture deficit of T3 caused greater stress to the crops.

The richness and diversity of the fungal community under the 10% reduced irrigation treatment were higher than those under the other treatments. While the richness and diversity of the fungal community under T3 were also higher, the dispersion between the community structure in T3 and other treatments was larger, showing a notable difference. Under the 10% reduced irrigation treatment or CK treatment, the nutrient metabolism capacity of the microbial community was higher than that under the other treatments, especially the N cycle, and the decomposition capacity of xylan and chitin was improved, which suggests that community catabolism was more active. The 10% reduced irrigation treatment increased soil CAT and SA activities, which may be related to the increase in communities such as Patescibacteria and Basidiomycota. The results of the structural equation model also show that different irrigation treatments can indirectly and significantly influence the yield of eggplants by affecting the microbial community structure, soil enzyme activity and soil nutrients, and the different irrigation treatments explained 70% of the variation in yield.

The regulation of photosynthesis is also an important factor that affects crop growth and yield (Wu et al., 2019). Studies have shown that using an improved irrigation method, alternate drip irrigation, can significantly increase the photosynthetic rate of crops and improve crop quality and yield (Wang et al., 2020). Many studies have shown that partial root zone irrigation can improve the crop transpiration rate (Du et al., 2006), increase the photosynthesis rate (Romero et al., 2012), and lead to the accumulation of more photosynthetic products (Shahnazari et al., 2007). Water deficit affects chlorophyll fluorescence parameters and pigments (Hazrati et al., 2016), photosynthetic electron transport and specific photoprotective responses (Zivcak et al., 2013). However, some soil microorganisms can protect the photosynthetic organs of crops under water deficit (Mathur et al., 2019) and can even increase the stomatal conductance compared with full watering (Augé et al., 2015), thereby improving the ability of plants to resist water loss (Rolli et al., 2015). This ensures that crop fruit quality and yield will not decrease and can even increase under relatively low irrigation. In our research, compared with full irrigation (CK), reducing the irrigation amount by 10% (T1) significantly increased the content of chlorophyll a in eggplant and increased the net photosynthetic rate, transpiration rate, stomatal conductance and water use efficiency.

In conclusion, our research proves that for greenhouse eggplants cultivated in autumn and winter, the optimal irrigation amounts at the seedling stage, flowering and fruit setting stage, full fruiting stage and harvesting stage are 180.43, 300.60, 405.97 and 315.76 m^3^ hm^−2^ 15 d^−1^, respectively. The 10% irrigation reduction (T1) treatment enhanced the richness and diversity of eggplant rhizosphere microbial communities and improved N cycling and nutrient catabolism, thereby increasing soil catalase, urease and alkaline phosphatase activities and improving soil nutrients. Plant-microbe interactions in the T1 treatment resulted in a significant increase in eggplant root length, surface area and vigor compared to those in CK. The contents of soluble sugar, soluble protein and free amino acids in fruit and the yield of eggplants were increased In T1, the bacteria Bacilli and the fungus Aspergillaceae were the most abundant, while the relative abundances of Chloroflexi and Acidobacteria were lower than those of the other treatments. However, in T3, the Basidiomycota (genus *Wallemia*) was the most abundant.

## Data availability statement

The datasets presented in this study can be found in online repositories. The names of the repository/repositories and accession number(s) can be found in the article/Supplementary material.

## Author contributions

FY, MW, and NW designed the study. TJ, XG, and FW carried out the experiments and analysed the data. PJ performed SEM analysis. TJ wrote the manuscript. FY, JL, and NW revised the manuscript. All authors contributed to the article and approved the submitted version.

## Funding

This work was supported by the National Key Research and Development Program (2019YFD1001904), the National Natural Sciences Foundations of China (NO. 32172556), and the Shandong Provincial Key Research and Development Program (2021LZGC017).

## Conflict of interest

The authors declare that the research was conducted in the absence of any commercial or financial relationships that could be construed as a potential conflict of interest.

## Publisher’s note

All claims expressed in this article are solely those of the authors and do not necessarily represent those of their affiliated organizations, or those of the publisher, the editors and the reviewers. Any product that may be evaluated in this article, or claim that may be made by its manufacturer, is not guaranteed or endorsed by the publisher.

## Footnotes

^1^ www.majorbio.com
