# Supplementary figures and images for: Proper irrigation amount for eggplant cultivation in a solar greenhouse improved plant growth, fruit quality and yield by influencing the soil microbial community and rhizosphere environment
Source: Front Microbiol. 2022 Sep 23;13:981288. doi: 10.3389/fmicb.2022.981288 (PMC9537383; doi:10.3389/fmicb.2022.981288)

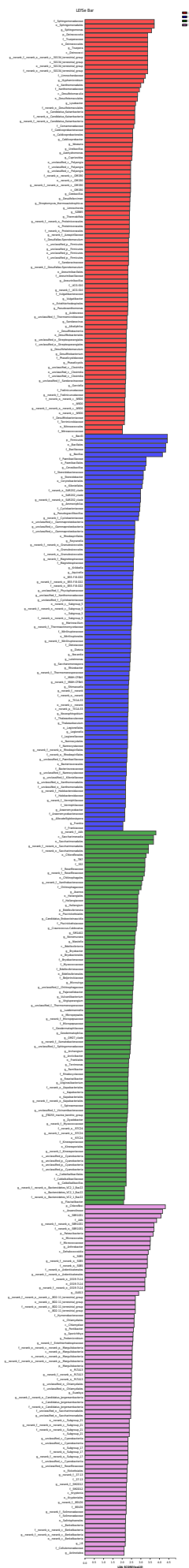

Supplement: Supplementary file 1 [file Data_Sheet_1.PDF]

LEfSe Bar

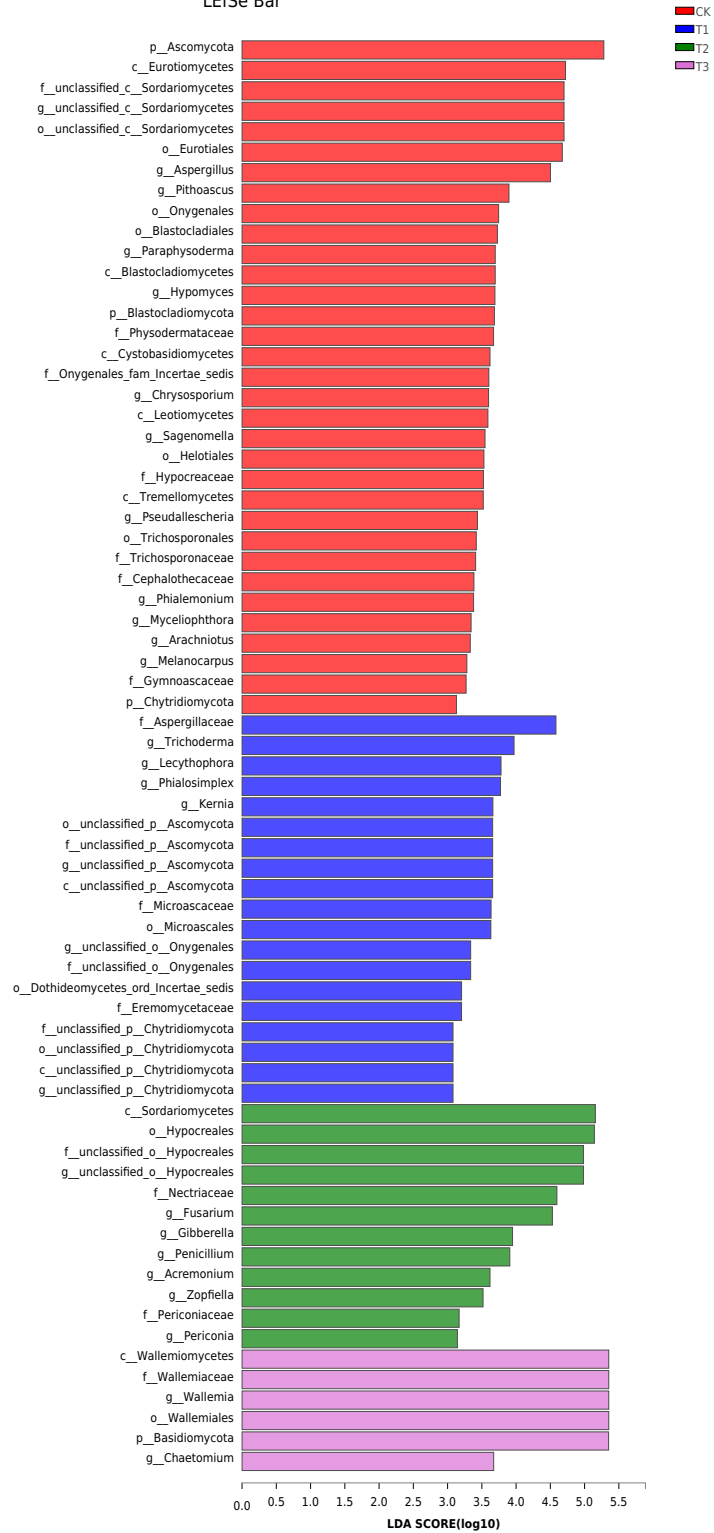

Supplement: Supplementary file 2 [file Data_Sheet_2.PDF]
