# Supplementary material for: Proper irrigation amount for eggplant cultivation in a solar greenhouse improved plant growth, fruit quality and yield by influencing the soil microbial community and rhizosphere environment
Source: Front Microbiol. 2022 Sep 23;13:981288. doi: 10.3389/fmicb.2022.981288 (PMC9537383; doi:10.3389/fmicb.2022.981288)
Supplement: Supplementary file 3 [file Data_Sheet_3.docx]

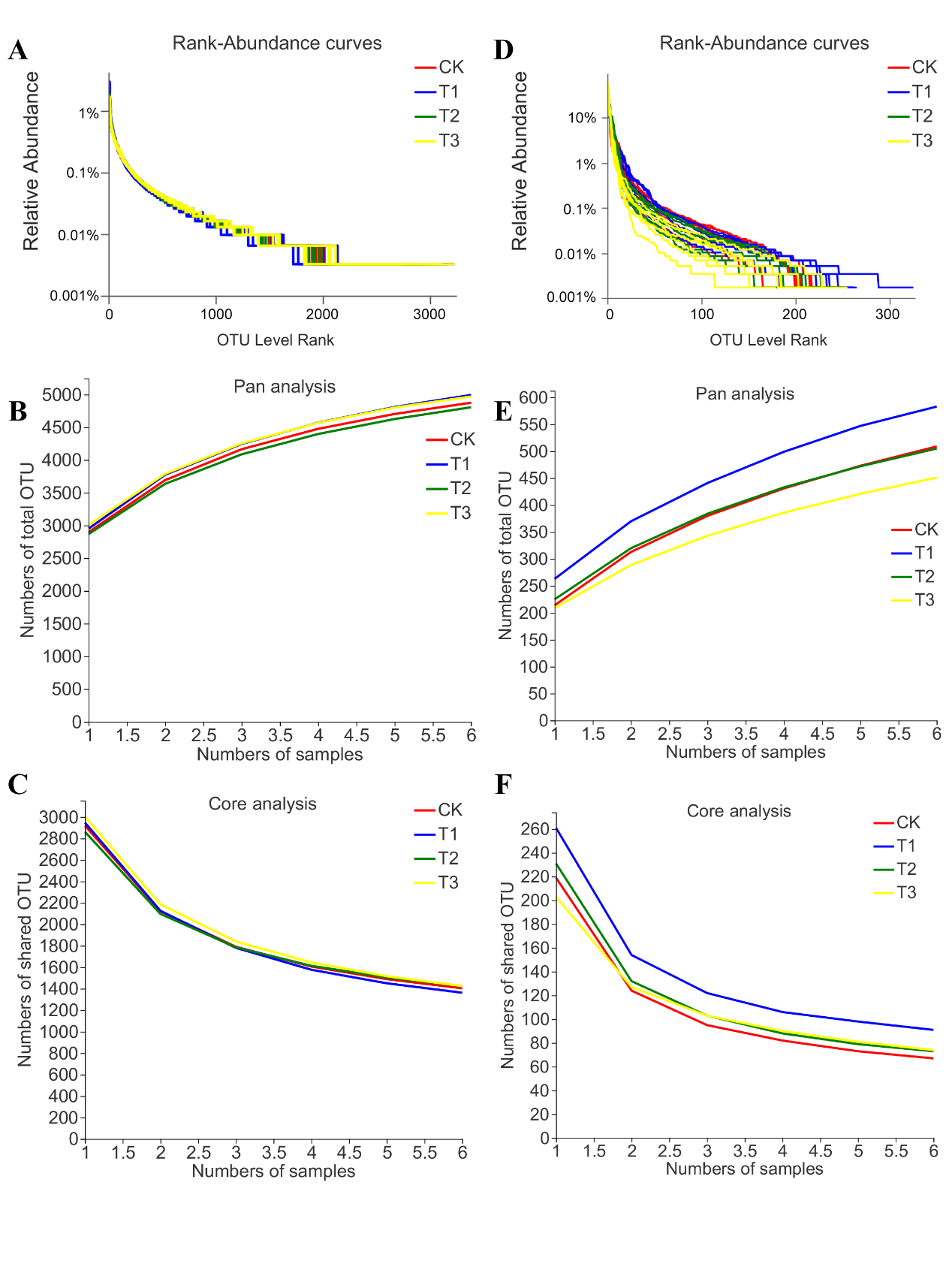


Fig. S1 Randomly extract the same number of OTU sequences from each sample, perform species taxonomic annotation on OTUs, statistical abundance information and display them as Rank-Abundance curves, including bacteria (A) and fungi (B). The Pan/Core analysis curve assesses whether the sample size for this sequencing is sufficient, including bacteria (B and C) and fungi (E and F). n=6.


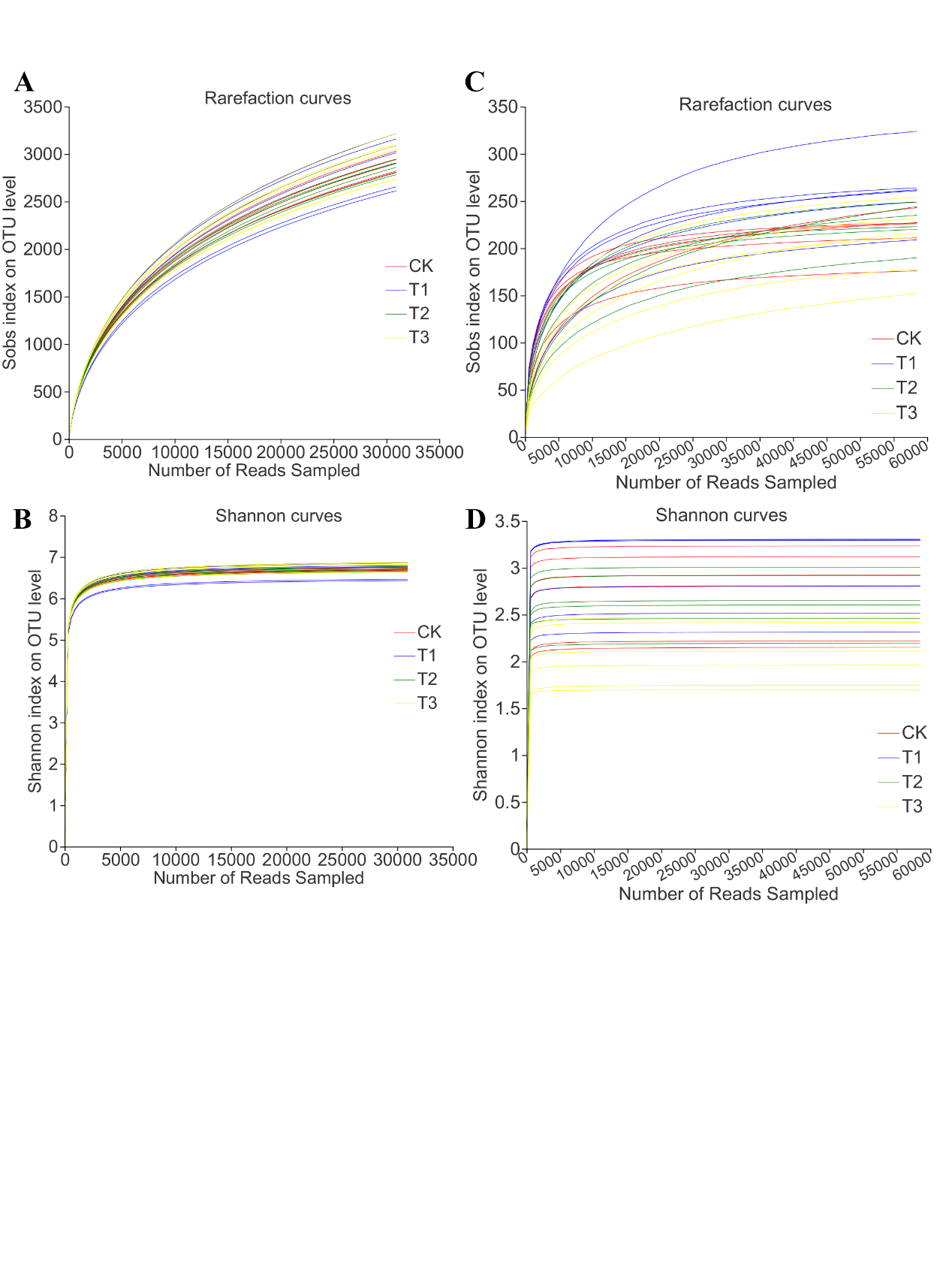
 Fig. S2 The Alpha diversity index assessment is based on randomly drawing the same number of OTUs in each sample, and the rarefaction curves for sobs and shannon are shown, including bacteria (A and B) and fungi (C and D). n=6.


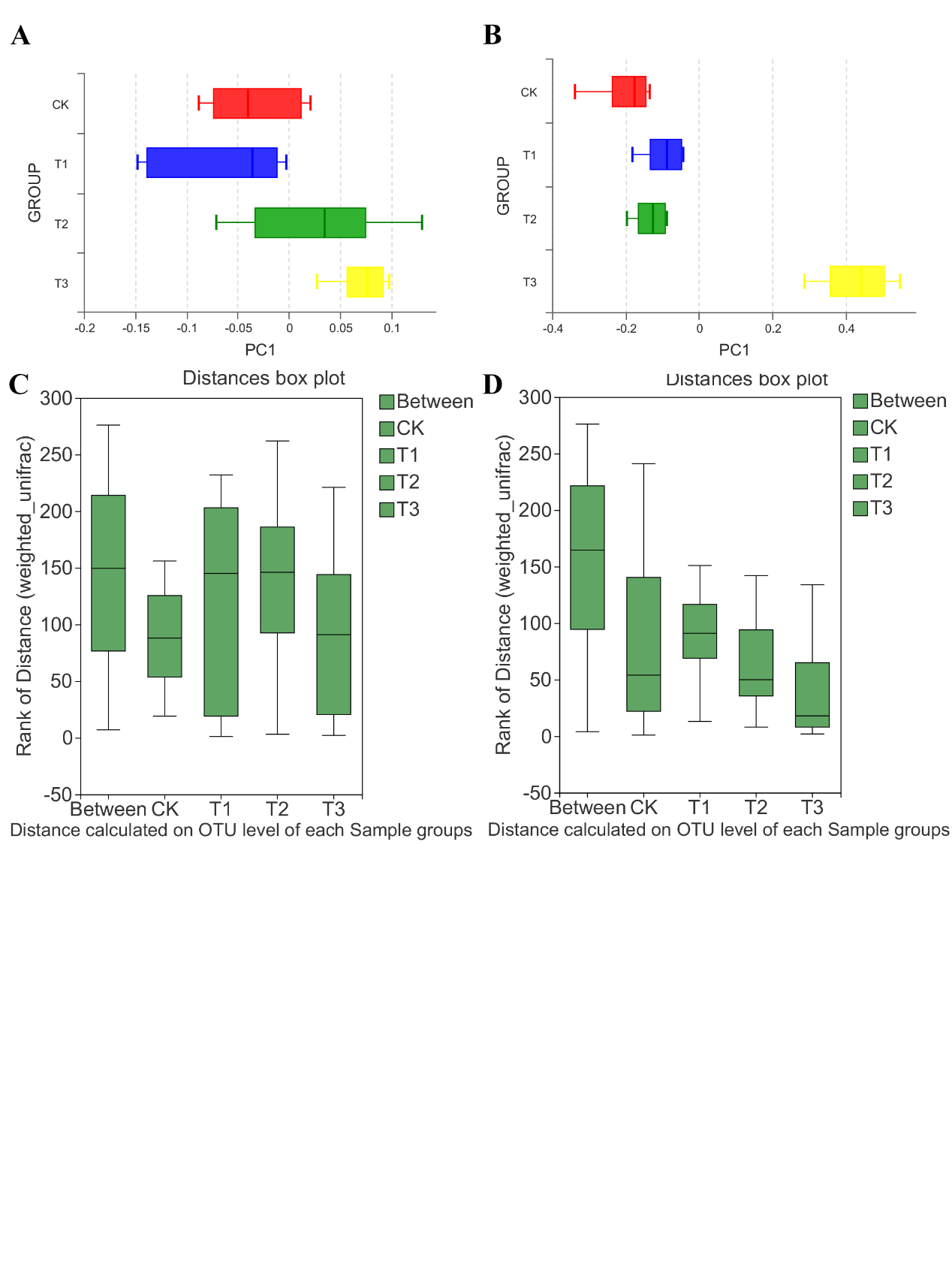


Fig. S3 The distribution of different treatments on the PC1 axis is discrete, including bacteria (A) and fungi (B). Analysis by ANOSIM/Adonis showed that the between-group differences (multi-group) of the different treatments were significantly greater than the within-group differences, including bacteria (C) and fungi (D) on OTU level with weighted unifrac distance algorithm. n=6.


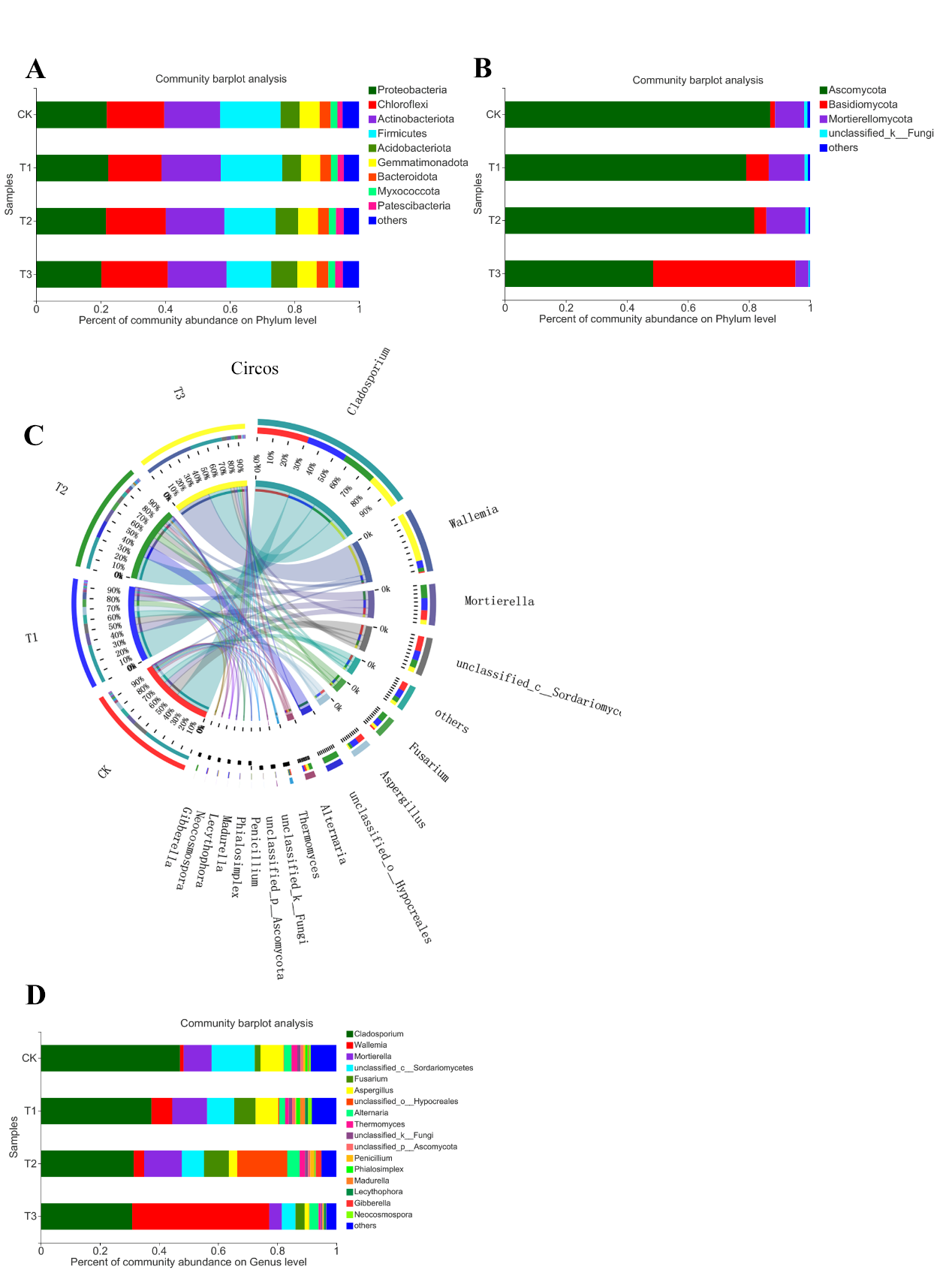


Fig. S4 Species composition of different treatments at each taxonomic phylum level, including bacteria (A) and fungi (B). Circos reflects the distribution ratio of dominant phyla in different treatments, and the distribution ratio of each dominant genus in different treatments of fungi (C). Species composition of different treatments at each taxonomic genus level of fungi (D). n=6.


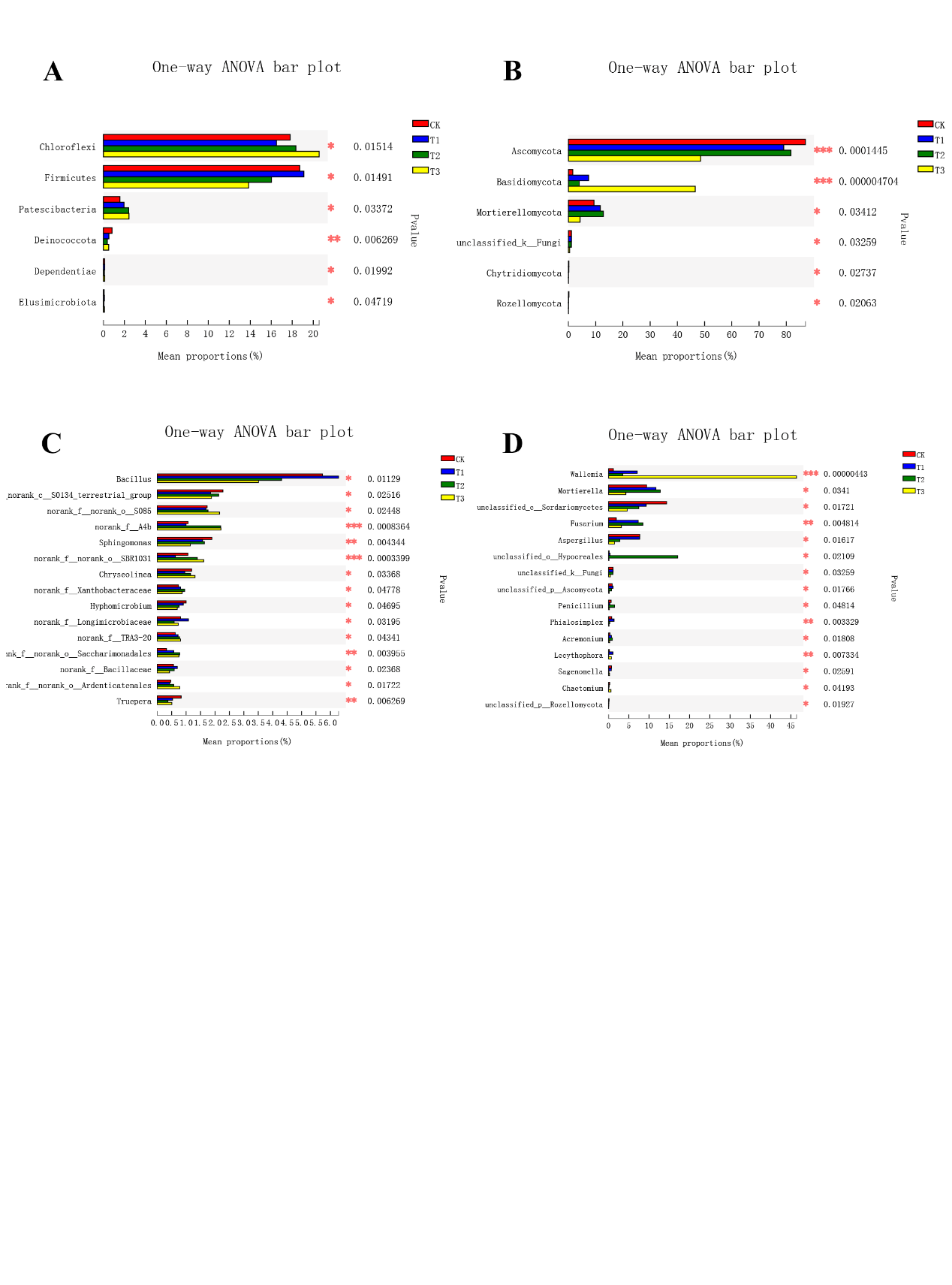


Fig. S5 Tests of significant differences between groups between different treatments, including bacterial phylum level (A), fungal phylum level (B), bacterial genus level (C) and fungal genus level (D). The test method is one-way ANOVA, the multiple test correction is fdr, and the post-hoc test is Tukey-Kramer, *P*<0.05. n=6.


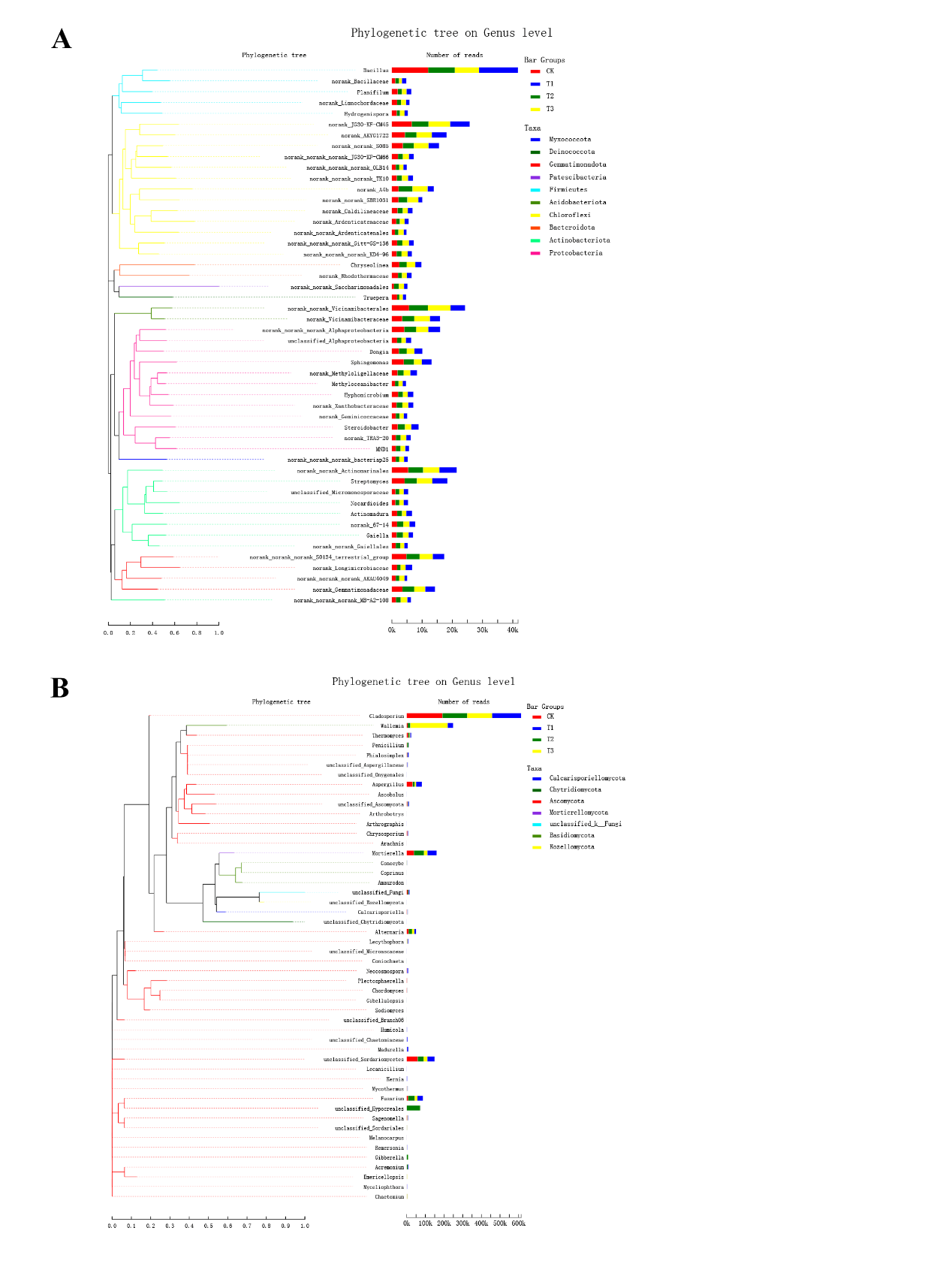


Fig. S6 Phylogenetic tree of bacteria (A) and fungi (B) under different treatments with number of reads on genus level. Different colors on phylogenetic tree means different phylum. n=6.
